# Arginine utilization in *Acinetobacter baumannii* is essential for pneumonia pathogenesis and is regulated by virulence regulator GacA

**DOI:** 10.1128/iai.00572-24

**Published:** 2025-04-02

**Authors:** Kuldip Devnath, Avik Pathak, Perwez Bakht, Ranjana Pathania

**Affiliations:** 1 Department of Biosciences and Bioengineering, Indian Institute of Technology Roorkee 30112 , Roorkee, Uttarakhand, India; University of Pennsylvania, Philadelphia, Pennsylvania, USA

**Keywords:** nosocomial infection, antibiotic resistance, arginine metabolism, BAL fluid

## Abstract

Nutrient availability in infection niches and the ability of bacterial pathogens to alter their metabolic landscape to utilize diverse carbon sources play a major role in determining the extent of pathogenesis. The vertebrate lung is rich in amino acids, such as arginine, which are available to the pathogens as a nutrient source to establish infection. Arginine is also used by the host nitric oxide synthase to synthesize nitric oxide, which is used against invading pathogens and for lung tissue repair. In this study, we have focused on the arginine catabolic pathway and its importance in the pathophysiology of *Acinetobacter baumannii*, a nosocomial pathogen, which is one of the major causes of ventilator-associated pneumonia, catheter-associated urinary tract infection, and so on. We show that the arginine succinyltransferase (AST) pathway is the predominant arginine catabolic pathway in *A. baumannii*. The genes of the AST pathway are arranged in an operon and are conserved in *Acinetobacter* spp. We show that the deletion mutant of the AST pathway failed to utilize arginine as a carbon source, and its virulence was severely compromised in an *in vivo* murine pneumonia infection model. We identified GacA as the positive regulator of the AST operon in *A. baumannii*, which is different from other bacterial pathogens. Our study highlights the importance of arginine utilization in the pathophysiology and virulence of *A. baumannii*. Owing to its importance in the pathophysiology of *A. baumannii*, the arginine catabolic pathway can further be investigated to assess its suitability as an antibacterial drug target.

## INTRODUCTION

The onset of infection largely depends on the ability of a pathogen to thrive and colonize the infection site, which is influenced by nutrient diversity and abundance (1, 2). Most pathogens possess an exceptional capability to alter their metabolic preferences and adapt to diverse host niches (1–4). This also helps to uncover conditionally essential pathways that can be further investigated for their suitability as drug targets (1–4). Most infection niches inside the human host contain free amino acids that can be catabolized by the invading pathogens as an energy source, enabling them to proliferate and establish an infection (3–5). The vertebrate lungs contain most of the free amino acids, which represent a nutrient pool readily available for the pathogens to thrive on (3, 6, 7). Some amino acids, such as arginine, are also important for the host (7–9). The host cells, through nitric oxide synthase (NOS), synthesize nitric oxide (NO) from arginine, which is used against invading pathogens and for lung tissue repair (10, 11). In addition, arginine is an essential component of the urea cycle in the host and is transformed into ornithine and urea, which regulate pH homeostasis (11).

*Acinetobacter baumannii* is a gram-negative nosocomial pathogen and one of the major causes of ventilator-associated pneumonia, urinary tract infections, meningitis, sepsis, and wound infections in hospitalized patients (12, 13). The metabolic landscape of *A. baumannii* and the underlying regulatory mechanisms in different infection niches remain largely unexplored. Although *A. baumannii* utilizes arginine (14), very little is known about its metabolism or contribution to pathogenesis. Previous studies on histidine and phenylacetic acid catabolism have demonstrated its importance in virulence and pathogenesis in *A. baumannii*, but the contribution of other amino acid catabolism is largely unknown (3, 15). *Pseudomonas aeruginosa* is able to outcompete the host for the utilization of valine and leucine, which has an adverse effect on the host’s energy metabolism and mitochondrial stress signaling (16). *P. aeruginosa* exhibits a chemotactic response to ornithine, a process that plays a crucial role in its pathogenesis and colonization (17). This demonstrates that amino acid metabolism significantly contributes to pathogenesis. With its plastic genome and the remarkable ability to acquire antibiotic-resistance genes, *A. baumannii* has developed resistance to most of the clinically relevant antibiotics (18, 19). Considering the contemporary antimicrobial resistance landscape, a deeper understanding of the niche-specific metabolic preferences of *A. baumannii* is important (1, 2, 6). This knowledge will significantly contribute to antibacterial drug discovery research, aiming to target crucial metabolic pathways in this multidrug-resistant pathogen.

Given the abundance of arginine in the lungs and its importance in the host defense, we sought to understand the arginine catabolic landscape and its importance in the pathophysiology of *A. baumannii*. In general, arginine is catabolized by bacteria via five major pathways, namely (i) arginase pathway, (ii) arginine deiminase (ADI) pathway, (iii) arginine succinyltransferase (AST) pathway, (iv) arginine transaminase (ATA)/oxidase/dehydrogenase pathway, and (v) the arginase decarboxylase (ADC) pathway (20). A previous study of *Streptococcus pyogenes* demonstrated that arginine catabolism proceeds via the ADI pathway, significantly contributing to virulence during the colonization of murine mucosa and playing a role in the host’s innate immune response (7). In *P. aeruginosa*, the AST pathway is the major arginine catabolic pathway under aerobic conditions (21). In *Escherichia coli* and *Salmonella Typhimurium*, under nitrogen-limiting conditions, the regulatory protein ArgR triggers the expression of the *astCADBE* operon to break down and use arginine as the only source of nitrogen (21). In *P. aeruginosa*, the ArgR protein does not show any sequence homology to the arginine regulatory proteins from enteric bacteria or *Bacillus subtilis*; rather, it belongs to the AraC/XylS family of transcriptional regulators (22). However, in *A. baumannii*, there is no report on the predominant arginine catabolic pathway, and the importance of arginine in the pathophysiology of *A. baumannii* has not been explored yet. We performed reverse transcription polymerase chain reaction (RT-PCR) to check the expression of representative members of individual arginine catabolic pathways in media containing arginine as the sole carbon source. The genes associated with the AST pathway were significantly upregulated, exhibiting markedly higher expression levels compared to the other pathway. A recent study in *A. baumannii* has highlighted the significance of the AST pathway involved in arginine utilization in maintaining membrane lipid composition and susceptibility to polymyxin (23). In addition to arginine, the AST pathway is also involved in the catabolism of ornithine, which is also present in the murine lungs (3, 24). However, the first step, succinylation, is mediated by ornithine succinyltransferase, encoded by the *astO* gene (25). We found that the AST pathway genes are expressed during an *in vivo* murine pneumonia infection, indicating the involvement of the AST pathway in arginine catabolism *in vivo*. We made a deletion mutant of the whole AST operon (Δ*astCADBE*), which rendered *A. baumannii* incapable of utilizing arginine as the sole carbon source. We then went on to assess the pathophysiological fitness of the *A. baumannii* ATCC 17978 VU (wild type) and the AST deletion mutant in an *in vivo* murine pneumonia infection model. Here, we found that the AST deletion mutant was severely compromised in virulence in the murine pneumonia model of infection. The AST deletion mutant also showed significant defects in attachment to A549 lung epithelial cells. Further, we have also identified GacA as a putative regulator of arginine catabolism. The *gacA* deletion mutant was unable to utilize arginine/ornithine as a carbon source, and the expression of the AST genes was downregulated in the *gacA* deletion mutant. This study in *A. baumannii* highlights the critical role of arginine catabolism by the AST pathway and its significance in lung pathogenesis.

## RESULTS

### *In vitro* and *in vivo* exposure to arginine induces the expression of AST operon genes

**Fig 1:**
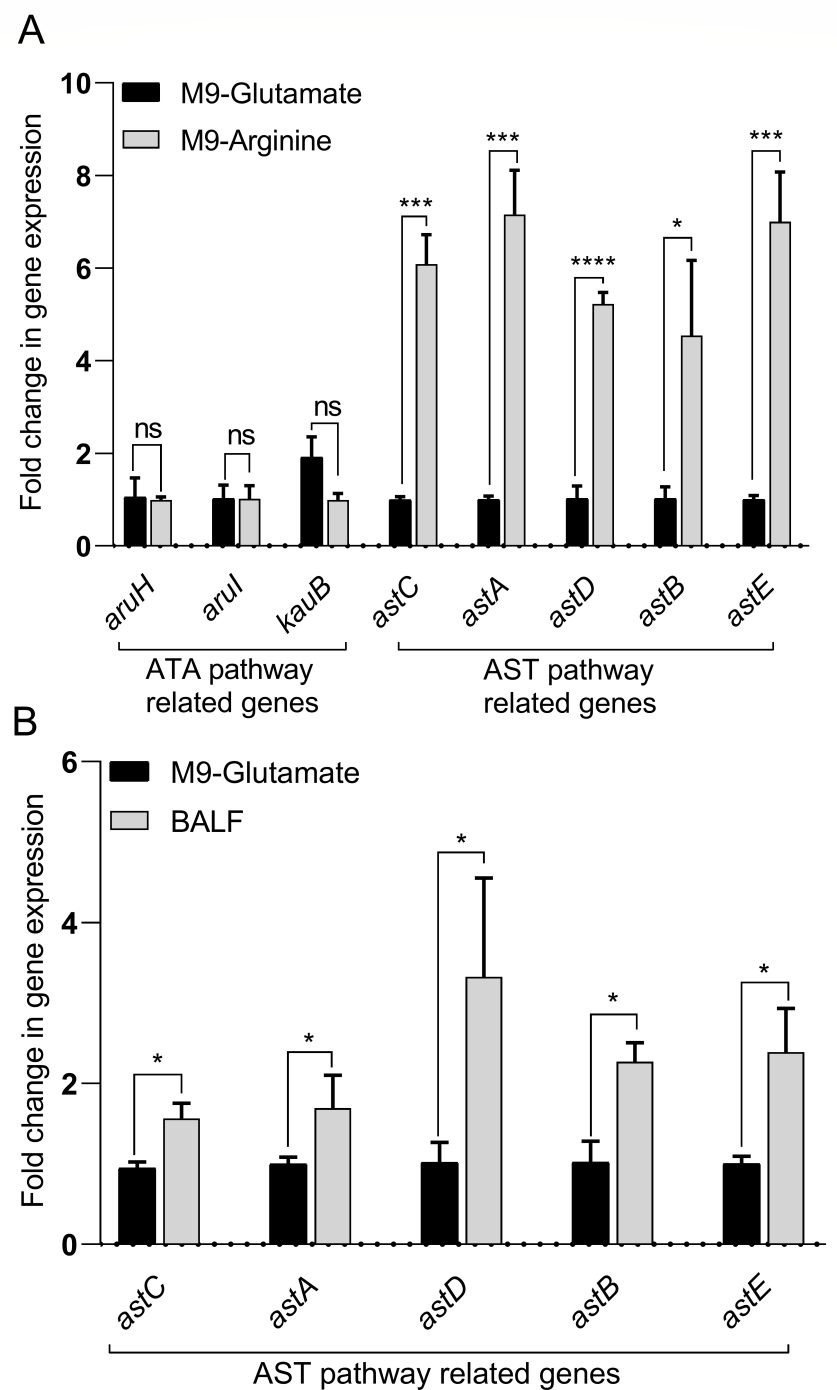
RT-PCR analysis of the *astCADBE* genes of *A. baumannii* showed enhanced expression in M9-arginine media and BALF of murine lungs. (A) *A. baumannii* wild-type cells were grown in M9 media supplemented with either glutamate or arginine as the sole carbon source overnight and then diluted 1:50 into fresh M9 media containing either glutamate or arginine as the sole carbon source. The cultures were incubated at 37°C with aeration for 12 h, followed by the extraction of total RNA. The expression of *astCADBE* genes in cells grown in M9-arginine was compared to cells grown in M9-glutamate. (B) To check the expression of ast genes under *in vivo* conditions, mice were infected with the wild-type cells and BALF was extracted 36 h post-infection. The expression of ast genes was compared to M9-glutamate control. The symbols ****, ***, and * represent *P* values of <0.0001, <0.001, and <0.05, respectively, while ns indicates no significant difference. The *rpoB* gene was used for normalization of gene expression using the ^ΔΔ^Ct method. The data presented represent the mean of three separate experiments. The error bars show the standard error relative to the control.

Bacterial pathogens with the ability to metabolize specific amino acids have a competitive fitness advantage during infection (1–3). In bacteria, there are five arginine catabolic pathways, as illustrated in Fig. S1 to S5. The protein sequences involved in the arginine catabolism pathways were retrieved from the NCBI database, and a thorough search led to the identification of the arginine catabolism pathways specific to *A. baumannii* ATCC 17978 VU (Methodology S1 to S5). We found that in *A. baumannii* ATCC 17978 VU, the ATA pathway is partially encoded, and the ADC, ADI, and arginase pathways are absent. In contrast, the complete AST operon is present, with all the annotated genes. The presence of a complete AST pathway and the partial ATA pathway in *A. baumannii* ATCC 17978 VU prompted us to investigate the expression of the genes encoding the enzymes of these pathways. We grew the wild-type *A. baumannii* strain in M9 minimal salts supplemented with 20 mM arginine or glutamate as the sole carbon source. Interestingly, we found that the expression of the AST pathway genes was the highest, suggesting that the expression of the AST pathway genes is responsive to exogenous arginine supplementation and may be important for arginine catabolism in *A. baumannii* (Fig. 1A). Arginine is present in bronchoalveolar lavage fluid (BALF) of mice (3), and to study the expression of AST pathway genes during *in vivo* infection, we isolated BALF from mice intranasally infected with the wild type. We checked the expression of the AST pathway genes in the BALF and compared it with the glutamate control. We found that the expression of the AST pathway genes was significantly upregulated in BALF (Fig. 1B). This further indicates that in the *in vivo* conditions, the AST pathway genes get upregulated, probably to enable *A. baumannii* to utilize arginine as a nutrient source.

### The AST pathway is present in *Acinetobacter* spp.

**Fig 2:**
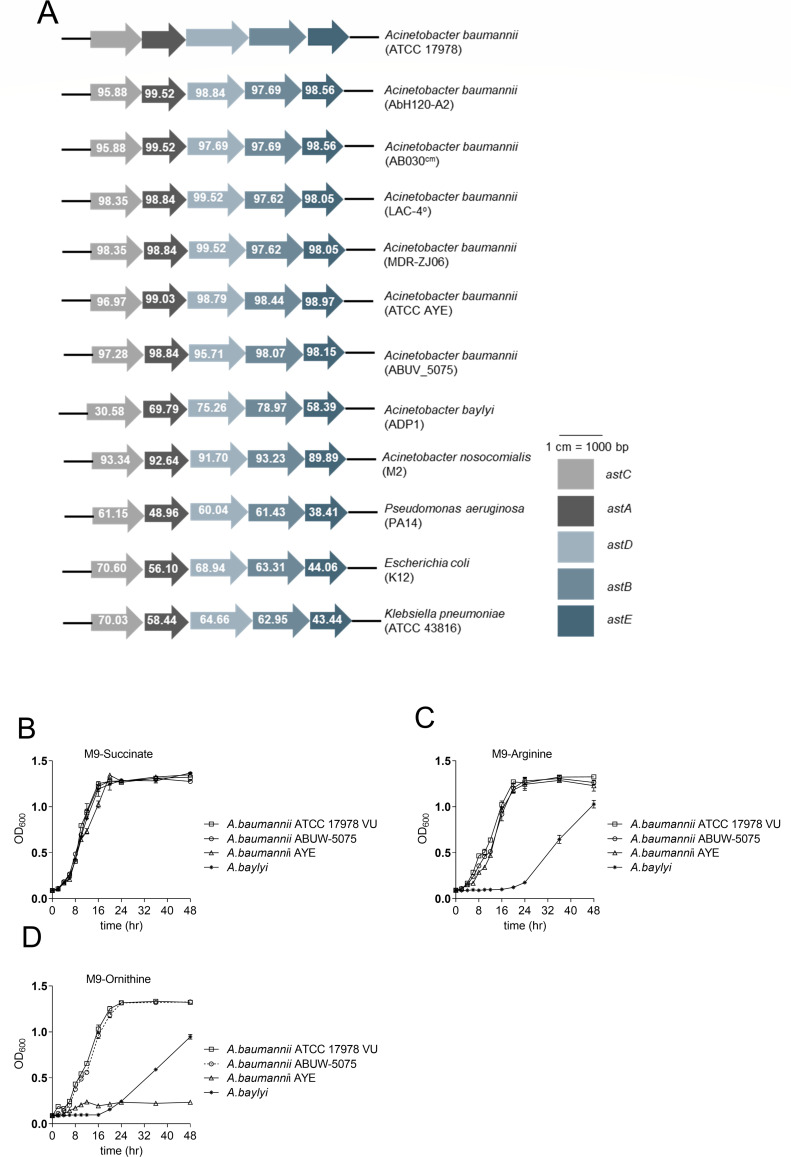
(A) Genomic organization of *astCADBE* operon in *Acinetobacter* spp., *Pseudomonas aeruginosa* PA14, *E. coli* K12, and *Klebsiella pneumoniae* ATCC 43816. The percentage of protein similarity relative to *A. baumannii* ATCC 17978 VU is indicated by the numbers inside the arrows. (B–D) To assess the ability of the pathogenic and environmental strains of *Acinetobacter* spp. to utilize arginine or ornithine as the sole carbon and nitrogen sources, the strains were grown in M9 minimal media. The cultures were incubated at 37°C with aeration for 48 h. For controls, succinate and NH_3_ were provided as the carbon and nitrogen sources, respectively.

Arginine is present in vertebrate lungs, and *A. baumannii* upregulates *astCADBE* genes in lung infection, so we investigated the organization and conservation of this operon. The *astCADBE* operon has five core enzymes that break down arginine to glutamate and succinate (Fig. S4). To check whether the AST genes are transcribed as a polycistronic mRNA, we performed a PCR with combinations of primers complementary to the upstream and downstream *astCADBE* genes using cDNA as the template. Using multiple combinations of these primers, we found that all the *astCADBE* genes lie in an operon (Fig. S6). We studied the relative conservation of the *astCADBE* operon in *Acinetobacter* spp. and other related species and used the *A. baumannii* ATCC 17978 VU strain as a template for all alignments. We found that the AST locus, *astCADBE*, is present in all the tested pathogenic strains of *A. baumannii*, sharing >93% amino acid similarity (Fig. 2A). We further assessed the ability of two representative pathogenic strains, ABUW5075 and AYE, and the environmental strain *Acinetobacter baylyi*, to utilize arginine/ornithine as the sole carbon and nitrogen source. We found that *A. baylyi* can utilize arginine/ornithine, although not as efficiently as the pathogenic strains *A. baumannii* ATCC 17978 VU, *A. baumannii* ABUW 5075, and *A. baumannii* AYE. (Fig. 2B and C). In *E. coli* and *P. aeruginosa*, the disruption of the *astC* gene impairs both arginine and ornithine catabolism, highlighting the importance of the AST pathway in arginine and ornithine catabolism (24, 26). This prompted us to investigate the role of the AST pathway in ornithine catabolism in *A. baumannii*. We found that the *A. baumannii* ATCC 17978 VU and *A. baumannii* ABUW-5075 strains could catabolize ornithine, but the *A. baumannii* AYE showed minimal growth in the presence of ornithine as the sole carbon and nitrogen sources. Since the *A. baumannii* AYE possesses the complete AST pathway and can catabolize arginine, the growth defect in the presence of ornithine as the sole carbon and nitrogen sources cannot be attributed to the AST pathway. In a recent study (25), the researchers showed that in *A. baumannii* ATCC17978, ornithine succinyl transferase, encoded by the *astO* gene, is involved in the first step, that is, succinylation of ornithine. Interestingly, we found that the *astO* gene is absent in *A. baumannii* AYE, which could be the reason behind the growth defect in the presence of ornithine as the sole carbon and nitrogen sources.

### The expression of AST operon is specific to arginine and is proportional to the arginine concentration in the medium

**Fig 3:**
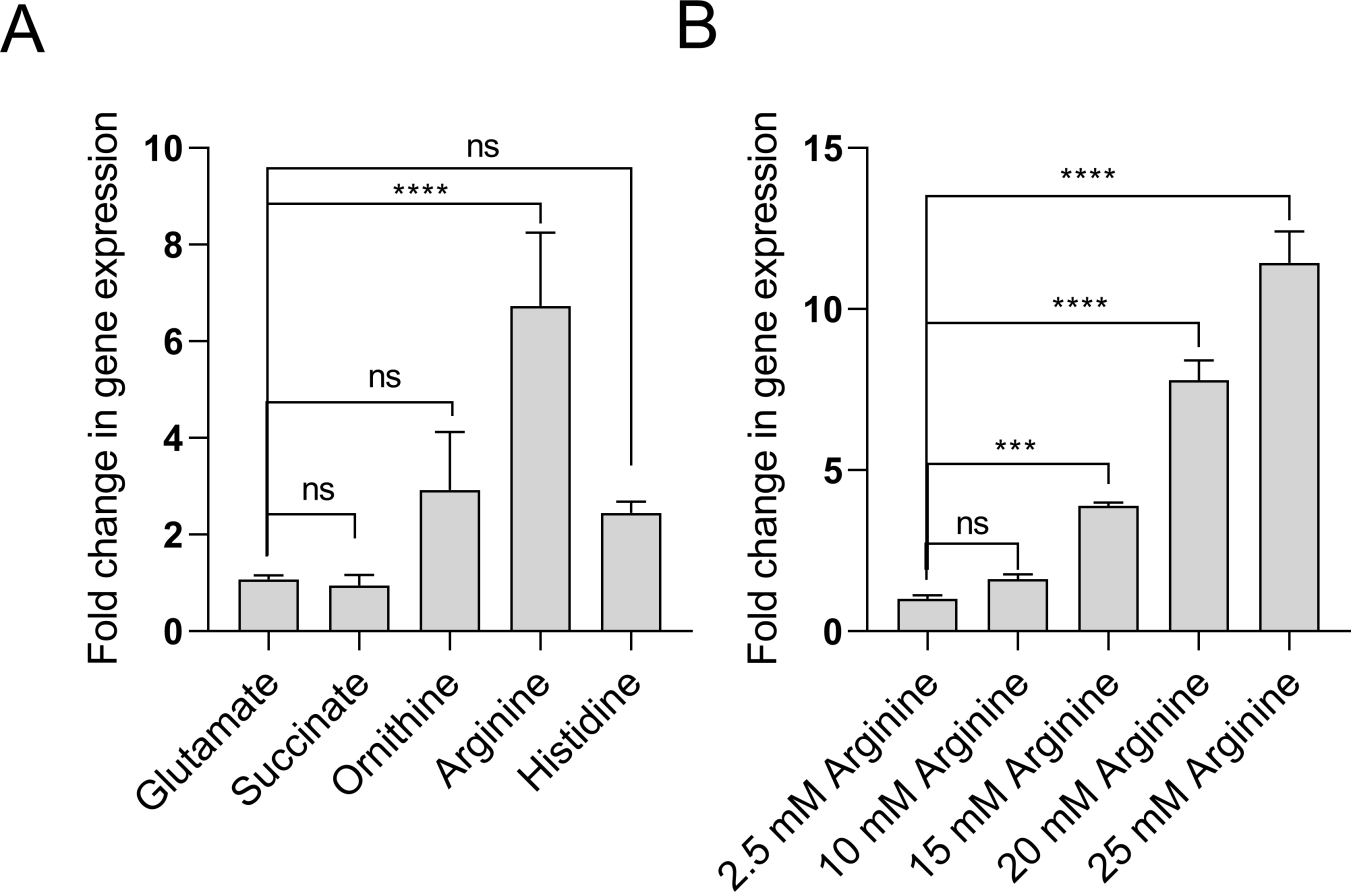
(A) The increase in arginine induces expression of the *astC* gene. Using 2.5 mM arginine as a control and a normalization of 1, the *astC* gene fold-change values are analyzed. (B) Arginine induces the expression of the *astC* gene compared to succinate, ornithine, and histidine. Glutamate was taken as the control, and relative fold change was determined. In both experiments, the wild-type overnight cultures, initially grown in M9 media with glutamate, were diluted 1:50 into fresh M9 media containing the desired carbon source. These cultures were then incubated at 37°C with aeration for up to 12 h, after which RNA was isolated. The data displayed are the average of three separate experiments, and the standard error concerning the control is indicated by the error bars. Statistical significance was determined using a one-way ANOVA followed by Dunnett’s multiple comparison test. The asterisks (**** and ***) denote *P* values <0.0001 and <0.001, respectively, while ns denotes no significant difference from the control.

To check whether the expression of the AST operon depends on the arginine content of the medium, we performed an RT-PCR analysis of the *astC* gene in cells grown in varying concentrations of arginine as the sole carbon source. We found that the expression of the *astC* gene is proportional to the arginine concentration in the medium (Fig. 3A). To further assess the specificity in the expression of the AST operon, we grew the wild-type cells in the presence of glutamate, succinate, ornithine, arginine, and histidine as the sole carbon source and checked the expression of the *astC* gene. We assessed the expression of the *astC* gene by comparing cells grown in glutamate as control to those grown with other carbon sources. The highest upregulation of the *astC* gene was observed when cells were grown with arginine as the sole carbon source (Fig. 3B). This demonstrates that the AST operon is specifically induced by arginine, highlighting its role in arginine catabolism in *A. baumannii*.

### The mutant strain Δ*astCADBE* cannot utilize arginine/ornithine.

**Fig 4:**
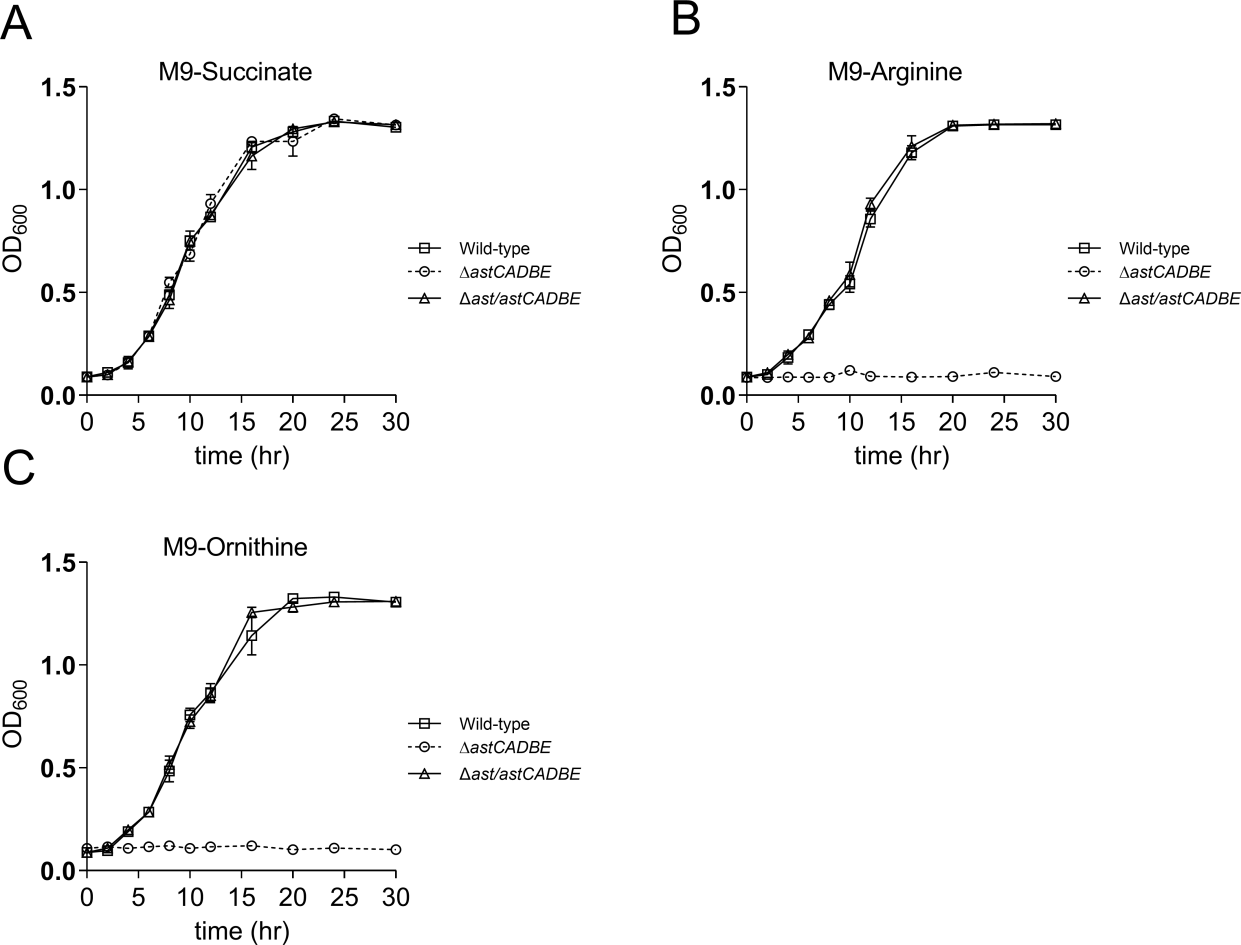
The *astCADBE* genes are critical for arginine utilization in *A. baumannii*. (A) Wild-type, Δ*astCADBE*, and the AST operon complemented strain Δ*ast/astCADBE* have no growth defects when grown in minimal media with succinate as the sole carbon source. (B and C) In minimum media containing only arginine or ornithine as their sole carbon source, the growth of the Δ*astCADBE* strain is compromised compared to wild-type and Δ*ast/astCADBE* strains.

To further assess the role of the *astCADBE* operon in arginine catabolism and the pathophysiology of *A. baumannii*, we generated a deletion mutant of the *astCADBE* operon, *∆astCADBE*, using a homologous recombination method (27). We also generated a complemented strain, ∆*ast/astCADBE*, using a similar homologous recombination method (27). We then performed a growth profile analysis of the wild-type strain, ∆*astCADBE*, and the ∆*ast/astCADBE* strains in either succinate, arginine, or ornithine as the sole carbon source. There was no growth difference in the presence of succinate as the sole carbon source (Fig. 4A). However, in the presence of arginine and ornithine as the sole carbon source, the ∆*astCADBE* mutant showed a severe growth defect. The growth defect was alleviated upon complementation (Fig. 4B and C). The primary role of the ADC pathway is to produce polyamines, which are essential for cell growth and function in *E. coli* and *P. aeruginosa* (21, 28). However, the ADC pathway contributes minimally to arginine catabolism in *E. coli*, with only about 3% of arginine consumption occurring through this pathway (24). In *P. aeruginosa*, the ADI pathway primarily provides ATP under anaerobic conditions (29). The ADI pathway is essential for *S. pyogenes*, enabling survival in nutrient-limited environments, such as during infection (7). *P. aeruginosa* and *E. coli* lack the arginase route for arginine catabolism, which has been well explored in *Bacillus spp* and *Helicobacter* spp. (20, 30). The first enzyme in the arginase pathway is not encoded in *A. baumannii,* which indicates that this bacterium does not utilize the arginase pathway for arginine catabolism. Our study revealed that wild type encodes a functional AST operon, whereas other arginine catabolism pathways, such as the ADC, ADI, and arginase pathways, are not present in this strain (Methodology S1 to S5). The ATA pathway is only partially encoded, missing the critical *gbuA* gene (Methodology S3). In *P. aeruginosa* PAO1, an AST pathway mutant can grow on arginine if functional ATA pathway genes are present (31). However, when the AST mutant also has a disrupted *gbuA* gene of the ATA pathway, the growth of arginine is abolished (31). In *A. baumannii*, the AST pathway is the only fully encoded pathway for arginine catabolism, which may account for the growth defect observed in the Δ*astCADBE* mutant when grown in M9 minimal media with arginine as the only carbon source. This suggests that the AST pathway is the primary route for arginine catabolism in *A. baumannii* under the conditions tested.

### Arginine catabolism is essential in *A. baumannii* for fitness during infection

**Fig 5:**
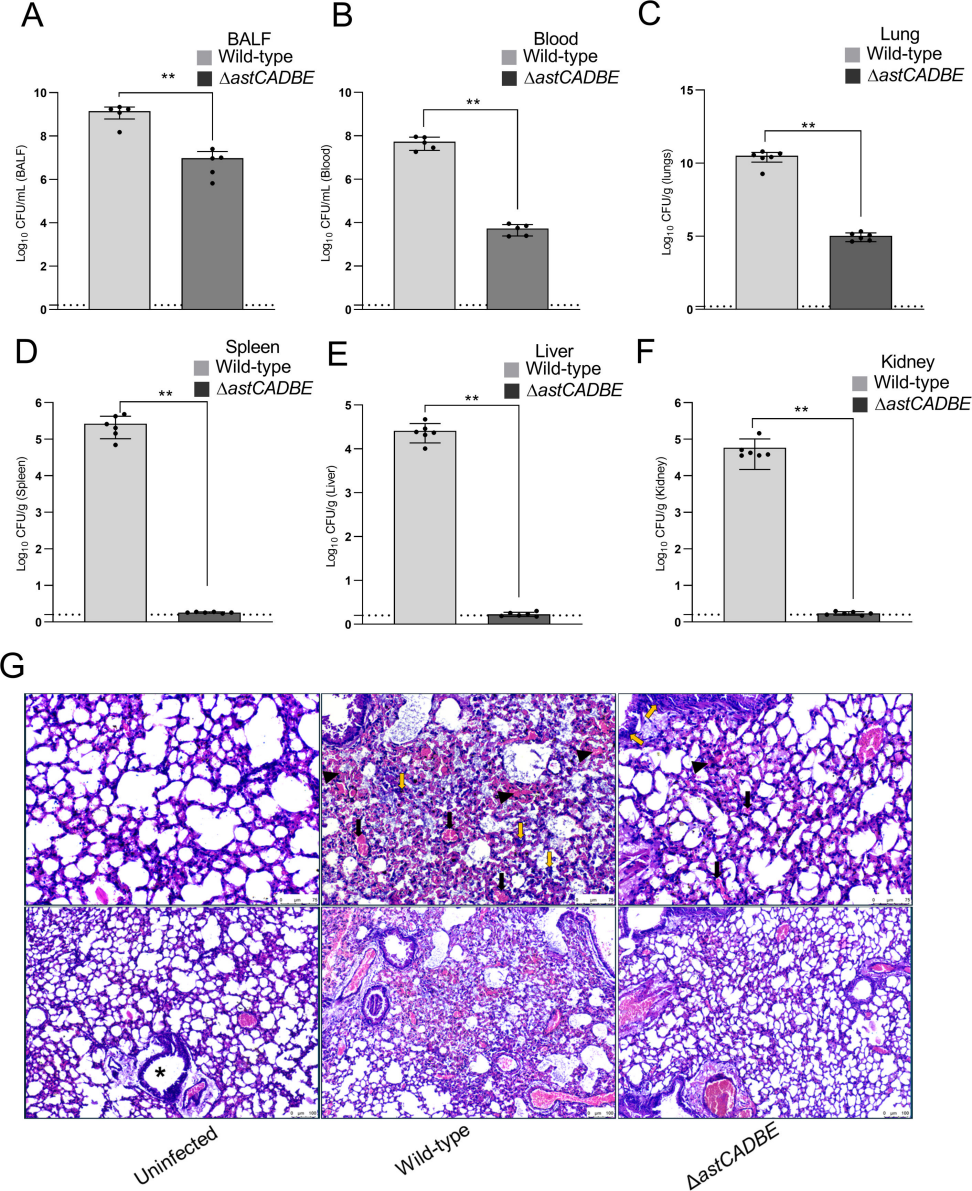
The *astCADBE* genes are critical for pathogenesis in a murine pneumonia infection. To determine bacterial burden in BALF and blood, two groups of mice (*n* = 5) were intranasally infected with wild-type and Δ*astCADBE* strains. Additionally, mice were divided into three groups (*n* = 6). This adjustment was done to assess bacterial burden in specific organs and requires tissue homogenization and histological tissue preparation, unlike BALF and blood collection, which require fewer samples. Murine lungs were intranasally infected with the wild-type, the Δ*astCADBE* strain, and physiological saline as a negative control. All the mice were sacrificed 36 h post-infection, and enumeration of bacterial burden was determined in wild type and Δ*astCADBE* at different infection sites: BALF (A), blood (B), lungs (C), liver (D), spleen (E), and kidney (F). Statistical significance was determined using Student’s *t*-test, with *<P* value <0.05 considered significant and *P* value <0.01 is indicated by **. (G) Representative histological images of hematoxylin and eosin (H&E) stained lung tissue infected with wild-type *Acinetobacter baumannii* and the mutant strain Δ*astCADBE*, uninfected lung served as control. The lower panel shows a low-magnification images with a 100 µm scale bare, while the upper panel shoes a high-magnification images of the same, with a 75 µm scale bar. The photomicrograph of the uninfected control reveals normal alveolar spaces lined by alveolar epithelium, with minimal hemorrhage and no increase in inflammatory cells. The bronchiole lumen (*) appears intact and undamaged. The wild-type infected lung tissue shows a loss of alveolar epithelial integrity, accompanied by increased inflammatory infiltration consisting of polymorphs and lymphocytes. Severe necrosis (indicated by the yellow arrow), congestion (indicated by the black arrow), and hemorrhage (indicated by the black triangle) are also observed in the wild-type infected lung tissue. The photomicrograph of lung tissue infected with the *A. baumannii* Δ*astCADBE* mutant strain reveals alveolar spaces with focal areas of hemorrhage and inflammation. Compared to the wild-type infected lung tissue, the mutant strain shows reduced levels of necrosis, congestion, and hemorrhage.

To assess the pathophysiological fitness of the ∆*astCADBE* strain, we performed an *in vivo* murine pneumonia infection study. First, we induced neutropenia in BALB/c mice using cyclophosphamide and then infected them intranasally with 5 × 10^8^ colony-forming unit (CFU) in 20 µL phosphate-buffered saline (PBS). The ∆*astCADBE* strain was severely compromised in virulence with a significantly lower bacterial burden in the BALF, blood, and lung tissue compared to the wild-type infected mice. In addition, the ability of the ∆*astCADBE* to spread to other organs, such as the liver, spleen, and kidney, was also significantly compromised (Fig. 5A through F). The histopathology study of the lung tissue revealed alveolar epithelial damage, severe necrosis, congestion, hemorrhage, and intense inflammatory infiltration in mice infected with the wild-type strain. In contrast, lung tissue from mice infected with the Δ*astCADBE* strain showed reduced damage, with focal hemorrhage and inflammation (Fig. 5G). We also performed an attachment assay to A549 lung epithelial cell line with the strains wild-type, Δ*astCADBE*, and Δ*ast/astCADBE* and found that the Δ*astCADBE* strain has a significant decrease in attachment as compared to the wild-type strain. The defect in attachment was alleviated upon complementation (Fig. S10). These findings of the attachment were consistent with the mice model of pneumonia. The results showed that arginine catabolism plays a significant role in virulence and lung colonization.

### Arginine concentration increases in BALF during *A. baumannii* pneumonia

**Fig 6:**
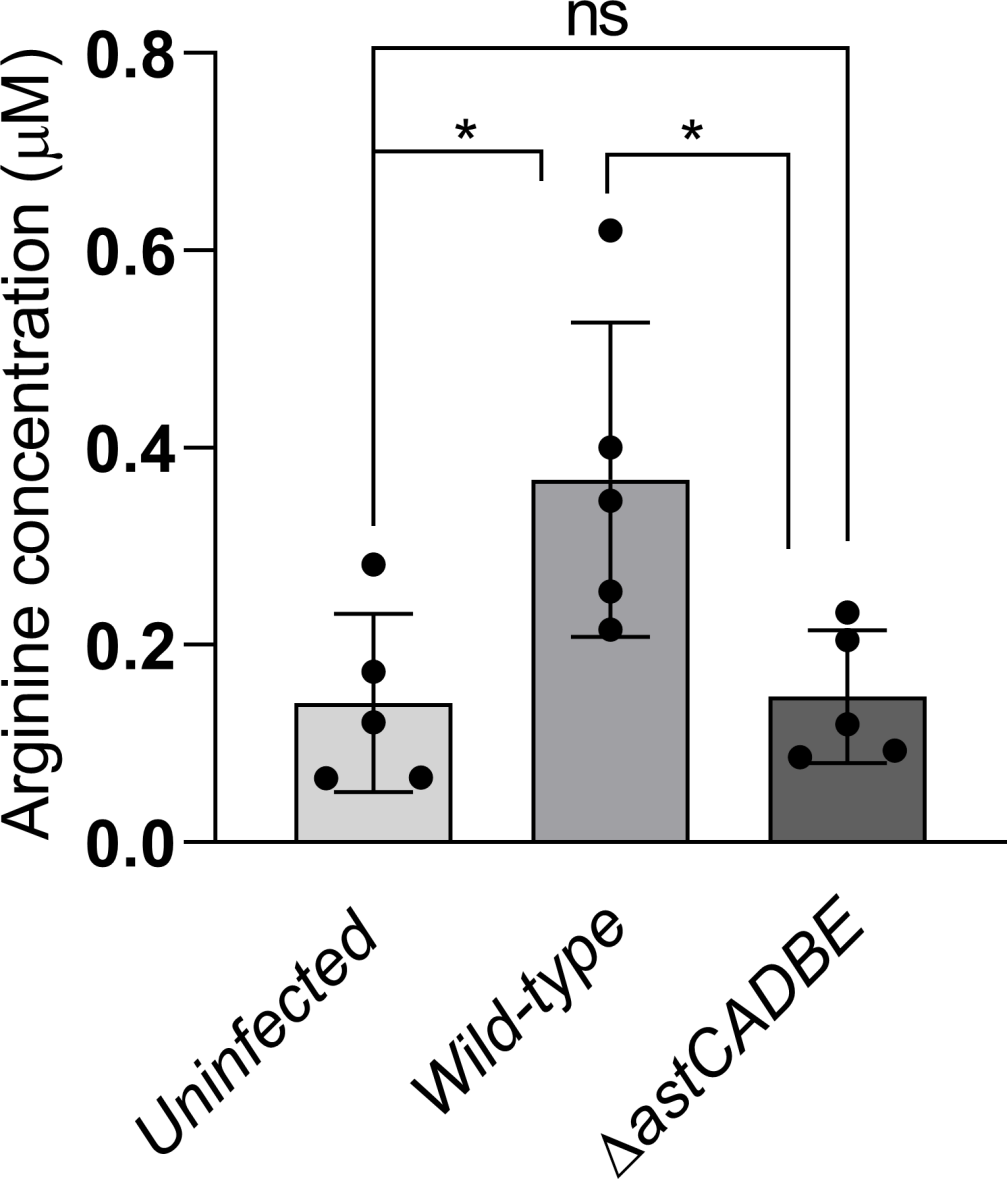
Arginine is available in the BALF of murine lungs. The wild-type infected strain had a higher concentration of arginine compared to the uninfected and Δ*astCADBE* infected strains, which had similar concentrations. To determine arginine in BALF, mice (*n* = 5) were intranasally infected with normal saline control, Δ*astCADBE,* and wild-type strain. A one-way ANOVA test along with Dunnett’s multiple comparison test was perfomed. The asterisk (*) denotes a *P* value of <0.05, whereas ns indicates no significant difference from the control.

To determine if *A. baumannii* infection alters arginine levels in the lung environment, we analyzed BALF samples from mice infected with the wild-type and Δ*astCADBE* strains, 36 h post-infection, alongside uninfected controls. Arginine level was higher in mice infected with the wild-type compared to the uninfected mice and the mice infected with the Δ*astCADBE* strain (Fig. 6). Our histopathological analysis shows that wild-type infection caused severe lung tissue damage compared to the Δ*astCADBE* mutant. This tissue damage likely resulted in the release of intracellular components (32), such as arginine, into the extracellular space, which may explain the increased arginine levels observed in the lungs infected with the wild-type strain. A previous study suggests that during pulmonary infections, such as those caused by *Mycobacterium tuberculosis*, arginine levels rise in the lungs, which is associated with enhanced immune responses (33). This suggests another potential cause for increased arginine levels in the lungs infected with the wild-type strain. The host produces NO using arginine as a substrate, via NOS enzymes found in the alveolar epithelial cells, macrophages, and other immune cells (34, 35). NO plays a critical role in immune responses by promoting the secretion of pro-inflammatory cytokines like TNF-α and IL-1β, which enhance host defense mechanisms against pathogens (36). NO also interacts with signaling pathways involved in the production of antimicrobial peptides, aiding in infection control (37). Additionally, NO supports lung tissue repair by promoting vasodilation, improving oxygenation, and modulating inflammation, which is essential for recovery following infection or injury (38). A previous study demonstrated that during lung infection with *P. aeruginosa*, arginine concentration increases in the lungs (39). This finding indicates that the infection modifies arginine levels in the lungs, which aligns closely with the results of our findings.

### The response regulator GacA positively regulates the AST operon in *A. baumannii*

**Fig 7:**
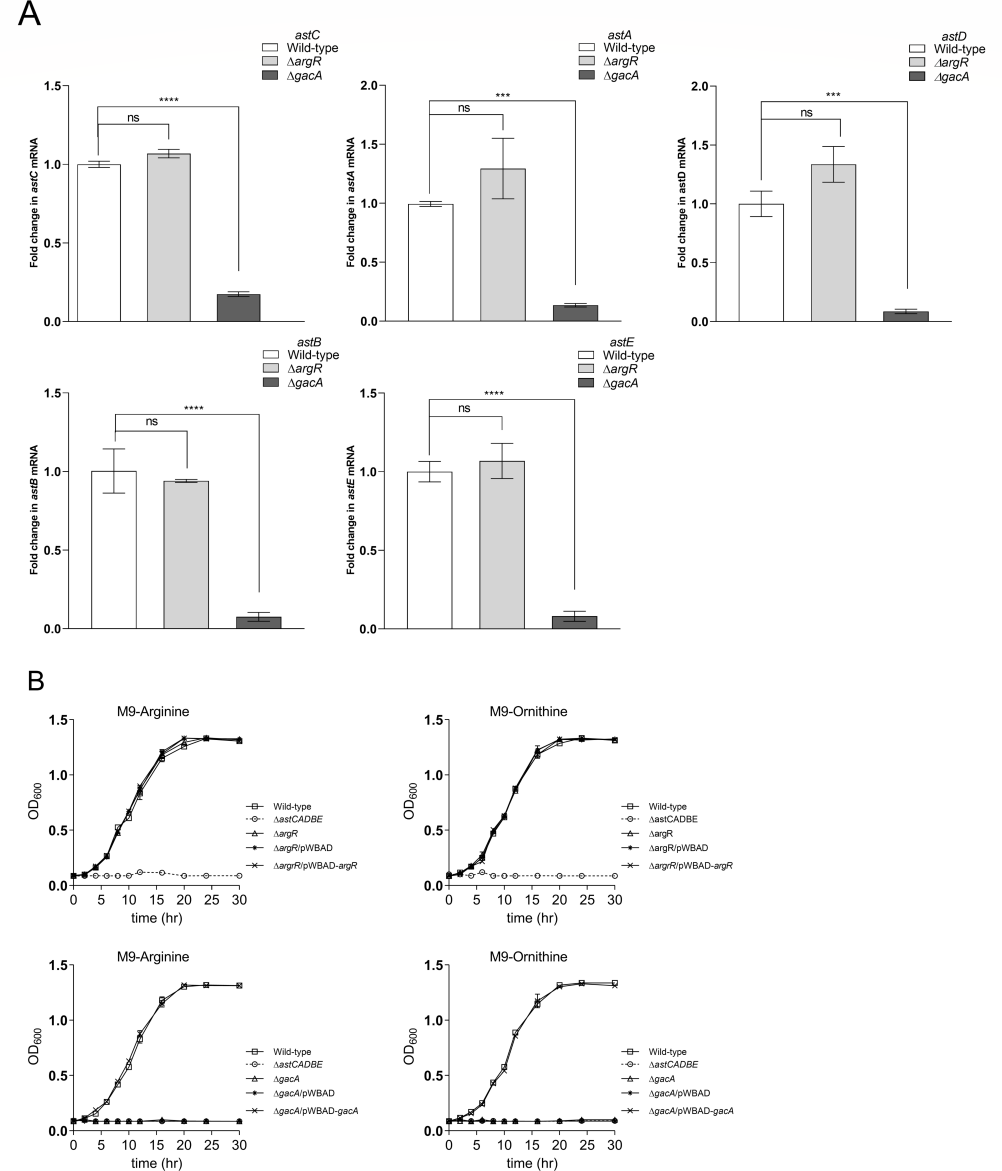
The response regulator GacA positively regulates the *astCADBE* operon. (A) The expression of *astCADBE* genes is altered in the Δ*gacA* strain in comparison to the wild-type and Δ*argR* strains. The data shown are the mean of three different experiments, and the error bars represent the standard error relative to the wild type. Statistical significance was determined by applying the one-way ANOVA and Dunnett’s multiple comparison test. The asterisks (**** and ***) denote *P* values of <0.0001 and <0.001, respectively, while ns indicates no significance. (B) *A. baumannii* Δ*gacA* exhibits impaired growth in minimal media with arginine or ornithine as the sole carbon source, showing a growth pattern like that of the control strain *A. baumannii* Δ*astCADBE*. However, the wild-type and *A. baumannii* Δ*argR* strains do not display a growth defect when compared to Δ*gacA* and Δ*astCADBE* strains. Complementation of *gacA* on the pWBAD plasmid restored arginine/ornithine utilization. The pWBAD plasmid backbone was introduced into the *A. baumannii* Δ*gacA* and Δ*argR* knockout strains to ensure that observed effects were due to the gene of interest and not the presence of the plasmid itself. These results indicate that GacA, rather than ArgR, regulates the *astCADBE* operon. Using the OD_600_, the growth curve studies are monitored over time. ANOVA, analysis of variance; OD, optical density.

The regulatory protein ArgR regulates the AST operon in *E. coli* and *S. Typhimurium* for the catabolism of arginine as a nitrogen source under nitrogen-limiting conditions (21, 24). In *P. aeruginosa*, the ArgR protein does not show any sequence homology to the arginine regulatory proteins from enteric bacteria or *B. subtilis* (21, 22). Rather, it belongs to the AraC/XylS family of transcriptional regulators and is auto-induced in the presence of exogenous arginine (22). To gain insight into the regulation of the AST operon in *A. baumannii*, we performed a homology search for arginine regulatory proteins using templates from *E. coli* and *P. aeruginosa*. We did not find any protein in *A. baumannii* homologous to *E. coli* ArgR, but we identified a protein from the AraC/XylS family (AUO97_RS05120), termed here as ArgR, sharing 31.68% homology with *P. aeruginosa* ArgR. To assess the role of this putative regulator in arginine catabolism, we generated a deletion mutant in the wild-type strain, *A. baumannii* Δ*argR*. The gene expression and growth profile analysis revealed no significant changes in the *astCADBE* operon expression level of *A. baumannii* Δ*argR* compared to the wild type (Fig. 7A and B). This indicates that the putative regulator does not play any role in arginine catabolism in *A. baumannii*. In a previous study, the putative regulator GacA was reported, where the expression of the *astCADBE* genes was downregulated in the *gacA* deletion mutant (15). We generated a Δ*gacA* mutant in wild-type strain to assess its role in regulating the *astCADBE* operon and arginine catabolism. RT-PCR analysis revealed that the *astCADBE* genes were significantly downregulated in the Δ*gacA* strain compared to the wild-type strain (Fig. 7A). Growth profile analysis showed a significant growth defect in the Δ*gacA* strain when grown with arginine or ornithine as the sole carbon source (Fig. 7B). These results highlight GacA as a positive regulator of the *astCADBE* genes and arginine catabolism.

## DISCUSSION

Bacterial pathogens possess the ability to switch their metabolic preferences to facilitate the use of nutrient sources available in the infection niches (40). Different carbon and nitrogen sources, such as amino acids, sugars, fatty acids, and so on, are present in different host niches and are utilized by pathogens during an infection (41, 42). Some of these metabolic pathways are conditionally essential, only required during infection, and are dispensable during growth in rich media (1, 2). Since resistance against conventionally targeted pathways, such as protein synthesis, nucleic acid biosynthesis, and so on, has already been developed by the pathogens, the conditionally essential pathways are good candidates for investigation as drug targets (1, 2, 41, 42). Murine lung is a reservoir of amino acids that are available to the pathogens to use as a nutrient source (3). Arginine is present in the vertebrate lung and is used by the host to synthesize NO, which is used against invading pathogens (3, 7, 9, 10). Given the importance of arginine in the host and its potential use by the pathogens, we sought to unravel the arginine catabolic landscape in *A. baumannii* and its importance in the pathophysiology. Previous studies on *Helicobacter pylori* showed that the pathogen utilizes its arginase enzyme to deplete arginine, limiting NO production in host and enabling bacterial survival (43, 44). This indicates that the pathogen and the host are in competition over the use of arginine.

In our study, we found that the *astCADBE* genes in wild type respond to arginine levels, indicating their functional role in arginine utilization. The AST pathway is fully encoded and likely serves as the primary route for arginine catabolism under our test conditions, as the Δ*astCADBE* mutant failed to grow in minimal media with arginine as the sole carbon source. Using combinations of primers and cDNA as the template, we identified that the *astCADBE* genes lie in a single operon in *Acinetobacter* spp., which is also the case in other gram-negative bacteria (24, 45–47). Furthermore, our results show that *A. baylyi* can utilize arginine, but less efficiently than the pathogenic strains *A. baumannii* ATCC 17978 VU, ABUW-5075, and AYE. This suggests that pathogenic *A. baumannii* strains have evolved to utilize arginine more effectively. Additionally, the *astCADBE* operon is expressed in murine lungs, which suggests arginine or ornithine utilization *in vivo*. This observation gives us insight into the arginine utilization characteristics of pathogenic *A. baumannii* strains. We also show that arginine induces the AST operon, and given its presence in the lungs, we generated a knockout of the entire operon in *A. baumannii* ATCC 17978 VU to understand its role in pathogenesis. In the murine pneumonia model, the wild-type strain showed higher CFU counts in the BALF and lung tissue, indicating better survival, while the Δ*astCADBE* mutant had reduced CFU counts, reflecting lower virulence. The decreased in CFU load in the lung environment likely resulted in less systemic spread of the Δ*astCADBE* mutant to other organs compared to the wild-type strain. As per previous reports, arginine metabolism contributes to biofilm formation by promoting attachment to biotic and abiotic surfaces (48–52). In *P. putida* and *P. aeruginosa*, arginine was found to enhance the intracellular c-di-GMP levels, which are involved in the expression of structural components of the biofilm (48–51). In *S. pyogenes*, the ADI pathway catabolizes arginine and enhances biofilm growth by maintaining pH homeostasis (52). Studies indicate that arginine utilization enhances bacterial persistence on abiotic surfaces via structures like pili and fimbriae, which also facilitate attachment to host tissues (52–54). For instance, in *A. baumannii*, pili, and the Bap protein are crucial for biofilm formation on abiotic surfaces and play a vital role in effective host tissue colonization (53, 54). As arginine utilization impacts bacterial attachment and our Δ*astCADBE* mutant strain cannot utilize arginine, we investigated the attachment of the wild-type and Δ*astCADBE* mutant strains to A549 lung epithelial cell lines. The assay revealed reduced attachment of the Δ*astCADBE* mutant compared to the wild type. This result is consistent with findings from the mice pneumonia model, indicating that arginine catabolism is essential for virulence and lung colonization. Since the *astCADBE* deletion mutant has a defect in arginine catabolism, we hypothesized that in mice infected with the *astCADBE* mutant, the arginine concentration in BALF will be higher than that of the wild-type infected mice and will be available for the host cells. Interestingly, we found that the arginine concentration in the BALF of wild-type infected mice was higher than the Δ*astCADBE* infected mice. Our histopathological study shows that wild-type infection causes severe lung damage compared to the Δ*astCADBE* mutant. Lung tissue damage releases intracellular contents (32), including amino acids like arginine, into the extracellular space and the BALF. The increase in arginine levels in the lung correlates with our histopathological findings of increased tissue damage in wild-type lung infection, which may further increase arginine concentration in BALF. Previous reports revealed that infections caused by *P. aeruginosa* and *M. tuberculosis* increase arginine concentration in the lungs (33, 39). This is probably due to the host’s physiological response to make arginine available to the immune cells at the infection site (33). NO plays a critical role in immune responses by promoting pro-inflammatory cytokine secretion (35, 36). Additionally, NO aids lung tissue damage repair by promoting vasodilation, improving oxygenation, and modulating inflammation (38). This finding suggests that the infection alters arginine levels in the lungs, which is consistent with the results of our research. A previous study in *S. pyogenes* also demonstrated that arginine catabolism is essential for virulence during the colonization of murine mucosa and has a role in the host’s innate immune response (7). This virtue of alternate nutrition acquisition of arginine may aid in extended pathogenesis and survival advantage. Ornithine is generated by the mammalian host during the urea cycle (11), and it is also found in BALF (3). The AST pathway catabolizes both arginine and ornithine, which may offer this pathway an extra benefit in substrate utilization for *A. baumannii* during infection and colonization in the host. This study highlights the significance of arginine/ornithine catabolism in *A. baumannii*.

Arginine is critical for lung pneumonia pathogenesis, and regulation of this amino acid utilization at the host-pathogen interface might be a key element during infection. To understand the regulation of arginine utilization, we sought to identify the arginine regulatory protein in *A. baumannii*. The regulatory proteins for phenylacetic acid, histidine, and cysteine metabolism have been identified and shown to be important in virulence in *A. baumannii*, based on previous reports (3, 15, 55). In *A. baumannii*, the CsrA regulator is required for growth in the presence of amino acids and human urine (56). In *S. Typhimurium* and *E. coli*, the AST pathway is regulated by the ArgR regulator (24, 24), while in *P. aeruginosa*, the arginine regulator belongs to the AraC/XylS family and shows no sequence similarity to *E. coli* ArgR protein (21, 22). We identified a similar AraC/XylS-like protein in *A. baumannii*, and our study revealed that this regulator protein does not regulate the AST pathway in *A. baumannii*. A study showed that a GacA mutant of *A. baumannii* has decreased expression of the AST operon (15), which prompted us to investigate the role of the GacA regulator in arginine utilization. Our findings revealed that GacA is a positive regulator of the AST pathway, and it is distinct from other pathogens.

The emergence of antibiotic resistance, particularly in *A. baumannii*, poses a significant challenge in clinical settings (18). Carbapenems, once considered the last line of defense against multidrug-resistant bacteria, are increasingly ineffective due to the rise of carbapenem-resistant *A. baumannii* (18). Co-infection with carbapenem-resistant *A. baumannii* is a critical challenge in COVID-19 patients, significantly elevating mortality rates (57). A study revealed that *A. baumannii* infections rose during the COVID-19 pandemic, and inadequate infection control could increase the risk of nosocomial outbreaks (57). The scarcity of new antimicrobials for gram-negative bacteria like *A. baumannii* has driven interest in alternative strategies (15, 58). Since pathogens have developed resistance to traditionally targeted pathways like protein synthesis and nucleic acid biosynthesis, conditionally essential pathways are promising targets for drug development (1–3, 41, 42). *A. baumannii* thrives in niches like the lungs, where arginine is available. Inhibiting key enzymes in the AST pathway could reduce the bacteria’s ability to catabolize arginine, thereby impairing its growth and virulence. In conclusion, this study highlights the role of the AST pathway in *A. baumannii* pneumonia and GacA as a positive regulator of the AST pathway. Given its importance in the pathophysiology of *A. baumannii*, the AST pathway can further be investigated to assess its suitability as an antibacterial drug target.

## MATERIALS AND METHODS

### Strains and culture conditions

All the bacterial strains used in this study are listed in Table S1. Previous studies have identified two *A. baumannii* ATCC 17978 variants based on the presence of the 44 kb AbaAL44 locus (59). In our study, the *A. baumannii* ATCC 17978 VU variant was found, which lacks the locus AbaAL44, and all isogenic mutants were derived from this strain. Strains of *A. baumannii* were cultured in LB broth at 37°C. *E. coli* DH5α was used for cloning, and apramycin (TCI chemical, India) 20 μg/mL was supplemented to LB broth whenever required. For growth in minimal media, bacterial strains were grown in M9 minimal medium (Himedia, India) supplemented with 1 mM MgSO_4_, and 0.1 mM CaCl_2_. Succinate, arginine, and ornithine were added to the M9 medium as the sole carbon source to a final concentration of 20 mM, unless otherwise stated. To test amino acid utilization as the only source of nitrogen, M9 was prepared with 3 g/L KH_2_PO_4_, 0.5 g/L NaCl, 6.78 g/L Na_2_HPO_4_, 10 mM sodium succinate, 1 mM MgSO_4_, and 0.1 mM CaCl_2_ supplemented with 20 mM of arginine/ornithine. Overnight cultures grown in minimal media were diluted 1:50 into fresh M9 media with the desired carbon source to achieve an optical density at at 600 nm (OD_600_) of 0.5. The cells are washed three times in PBS and diluted 1:100 in M9 minimal media for growth pattern analysis by incubation at 37°C with aeration. OD_600_ was monitored at different time intervals.

### Bacterial mutant generation

A homologous recombination strategy was used to create the deletion mutants with some modifications (27, 60). PCR amplicon containing the apramycin cassette flanked by 150-bp upstream and downstream regions of the gene of interest was transformed into *A. baumannii* electrocompetent cells harboring pAT02 (contains RecAb). The recombinants were selected on LB agar plates supplemented with 20 μg/mL of apramycin. The apramycin cassette was later excised out using flippase (FLP)-FRT recombination by transforming a plasmid expressing an FLP) enzyme. The comparison of upstream and downstream gene expression between wild-type and knockout strains showed no significant differences, indicating no polar effects in the knockout (Fig. S8 to S10). For complementation of AST operon genes, the AST deletion mutant harboring pAT02 plasmid was transformed with PCR amplicon containing AST operon genes flanked by 150-bp upstream and downstream sequences. All cloning plasmids and primers used for complementation and gene deletion in this study are provided in Tables S2 and S3.

### Transcriptional linkage analyses of the AST operon

Overnight cultures were diluted 1:50 into fresh media and incubated at 37°C with aeration till OD_600_ of 0.5. RNA was extracted using the RNeasy Mini Kit (Qiagen) following the manufacturer’s instructions. The isolated RNA was treated with DNase1 (Thermo Fisher Scientific) to remove genomic DNA. The DNase1-treated RNA was reverse transcribed using Prime Script First-Strand cDNA Synthesis Kit (Takara) following the manufacturer’s instructions. Transcriptional linkage was determined using primer combinations as described earlier (61). All the primer sets used are described in Supporting Table S3.

### Quantitative RT-PCR

The cDNA were subjected to real-time PCR using SYBR Green master mix (Thermo Fisher Scientific) on a Quant Studio 5 system real-time thermocycler (Applied Biosystems). The expression of target genes was normalized with the expression of 16S and compared to a control using the ^ΔΔ^Ct method. All the relevant primers used are described in Supporting Table S3.

### Murine pneumonia model and extraction of BALF

Neutropenia was induced in BALB/c mice with cyclophosphamide at a concentration of 150 mg/kg mice body weight, and then, the mice were intranasally infected with the wild-type and Δ*astCADBE* strains with 5 × 10^8^ CFU in 20 µL PBS (60–63). The animals were sacrificed 36 h post-infection. The organs were harvested and washed with cold physiological saline. Further, the organs were processed for CFU determination and histopathological analysis. For CFU determination, the organs were homogenized and subjected to serial dilution followed by plating on LB agar plates. For histopathology studies, organs were fixed for 48 h in 10% formalin and placed in paraffin blocks for sectioning. Hematoxylin and eosin stains were used to stain tissue slices, and slides were examined under a microscope (Leica DM1000 LED). BALF was isolated 36 h post-infection as described previously (3). The bacterial load in BALF was determined using a similar serial dilution method. The BALF was centrifuged at 1,000 × *g* at 4°C for 15 min to remove cells. Further, the BALF supernatant was syringe filtered using a 0.22 µm filter and analyzed for amino acid quantification.

### Amino acid quantification from BALF

HPLC-1260 Infinity (Agilent) was used to quantify amino acids from BALF. The BALF samples were derivatized by adding borate buffer and AccQ Tag ultra reagent to the samples. Mobile phases A and B consisted of 0.1% formic acid in water and 0.1 % formic acid in acetonitrile, respectively. The samples were incubated at 55˚C for 10 min for the derivatization process. Following the derivatization, 1 µL was loaded onto the instrument and quantified using a Waters Amino Acid standard. The UV Chromatograms at 260 nm were further analyzed for quantification.

### Attachment assay

A549 human alveolar epithelial cells were used to assess the adhesion abilities of the wild-type and Δ*astCADBE* strains following a slightly modified protocol from the previous description (53, 62, 64, 65). Confluent monolayers of A549 cells were incubated at 37°C for 1 h after being infected with approximately 2 × 10^6^ bacterial cells. The A549-infected cells with bacteria were subjected to gentle five rounds of PBS washing. After the bacteria were allowed to interact with the A549 cells, five gentle washing steps were performed to remove any non-attached bacteria, ensuring that only the bacteria physically attached to the cell surface remained. Further cells were resuspended in 1 mL of PBS after being trypsinized and subjected to serial dilution. Unlike the typical invasion assay, which uses gentamicin to kill bacteria added to cell monolayers, our assay was conducted without any antibiotics. After serial dilution, the cells were plated on LB agar and incubated at 37°C for 18. The percentage attachment was calculated by dividing the CFU recovered post-infection by the initial CFU of the inoculum. Attachment to A549 cells was expressed as a normalized percentage, with the wild-type average set to 100 %.

### Sequence analysis

Sequence analysis was carried out using techniques from an earlier study (3). The *astCADBE* operon protein sequences of *A. baumannii* ATCC 17978 VU were retrieved from the NCBI protein database. To find homologs of the AST operon in *A. baumannii* and other bacteria, the Basic Local Alignment Search Tool was used. The retrieved protein sequences were further analyzed for percentage protein similarity.

### Statistical analyses

GraphPad Prism 8 program and Microsoft Excel were used to conduct statistical analyses. All the statistical tests, group sizes, and independent experiment replicates are described in the figure legend. The *P* values were considered significant at ≤0.05.

## SUPPLEMENTAL MATERIAL

Supplemental material iai.00572-24-s0001.docx Fig. S1 to S10; Tables S1 to S3; Supplemental methods.

ASM does not own the copyrights to Supplemental Material that may be linked to, or accessed through, an article. The authors have granted ASM a non-exclusive, world-wide license to publish the Supplemental Material files. Please contact the corresponding author directly for reuse.
