# Supplementary material for: Arginine utilization in Acinetobacter baumannii is essential for pneumonia pathogenesis and is regulated by virulence regulator GacA
Source: Infect Immun. 2025 Apr 2;93(5):e00572-24. doi: 10.1128/iai.00572-24 (PMC12070743; doi:10.1128/iai.00572-24)
Supplement: Supplemental material — Fig. S1 to S10; Tables S1 to S3; Supplemental methods. [file iai.00572-24-s0001.docx]

**Supplementary file**

Arginine utilization in *Acinetobacter baumannii* is essential for pneumonia pathogenesis and is regulated by virulence regulator GacA

Kuldip Devnath^1^, Avik Pathak^1^, Perwez Bakht^1^, Ranjana Pathania^1*^

^1^Department of Biosciences and Bioengineering, Indian Institute of Technology Roorkee, Roorkee 247667, India.

^*^Corresponding author: Professor Ranjana Pathania, Department of Biosciences and Bioengineering, Indian Institute of Technology Roorkee, Roorkee 247667, India.

Email: ranjana.pathania@bt.iitr.ac.in

**Supplementary figures**


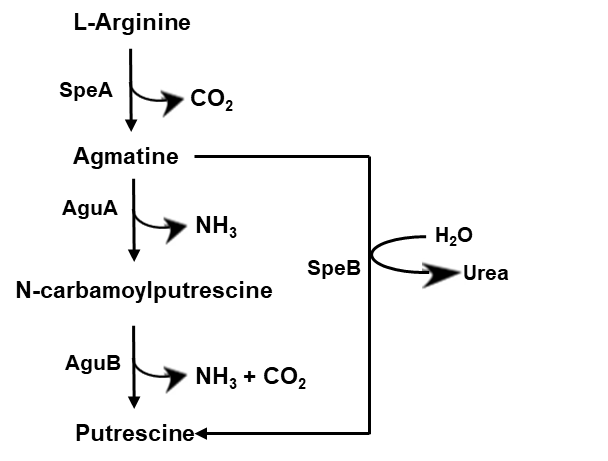


**Figure S1**. Schematic representation of the arginine decarboxylase (ADC) pathway. This pathway produces polyamines. Arginine decarboxylase (*speA*), the first enzyme in this pathway, converts arginine to agmatine. Further, agmatine can be converted to putrescine in two steps by agmatine deiminase (*AguA*) and N-carbamoyl putrescine amidohydrolase (*AguB*), or in one step via agmatine ureohydrolase (*SpeB*, agmatinase). The product putrescine can be catabolized to succinate semialdehyde, but the main function of this ADC pathway is to produce polyamines instead of breaking down arginine as a source of nutrients (1-3).


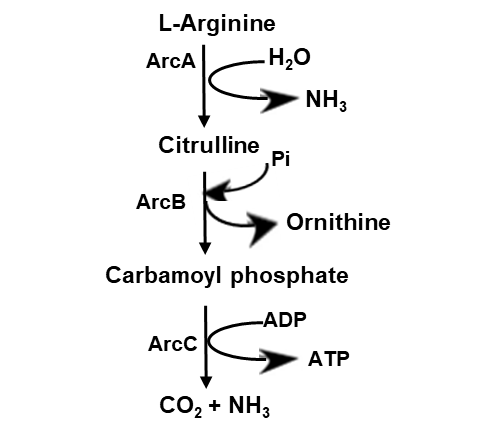


**Figure S2.** Schematic representation of the arginine deiminase (ADI) pathway. Genes of the ADI pathway was first characterized in *Pseudomonas aeruginosa* as the *arcDABC* operon and the major physiological function of this pathway is to supply ATP (4,5). Ornithine trans-carbamoylase (OTC), carbamate kinase (CK), and arginine deiminase are the three important ADI pathway enzymes that are encoded by the genes *arcB*, *arcC*, and *arcA*, respectively. The membrane protein arginine/ornithine antiporter (ArcD), which is encoded by the gene *arcD*, permits the simultaneous transport of arginine into the cell and ornithine out.


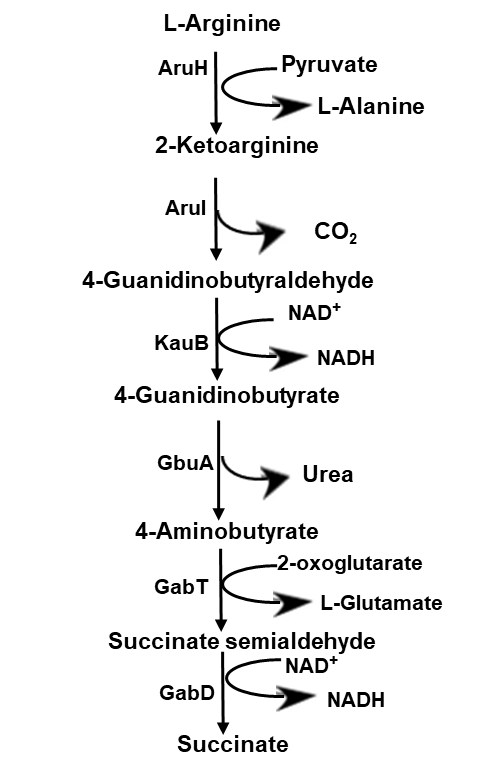


**Figure S3.** Schematic representation of the arginine transaminase (ATA) pathway. The pathway was characterized in *P. aeruginosa* (1,6). In this pathway arginine is catabolized into succinate. The primary enzyme involved in the ATA pathway is arginine pyruvate transaminase (AruH), which catalyses the reaction between L-arginine and pyruvate to produce 2-ketoarginine and L-alanine. 2-ketoarginine is further converted to succinate by the enzymes 2-Ketoarginine decarboxylase (AruI), 4-Guanidinobutyraldehyde dehydrogenase (KauB), guanidino butyrase (GbuA), 4-Aminobutyrate transaminase (GabT) and succinate semialdehyde dehydrogenase (GabD) respectively.


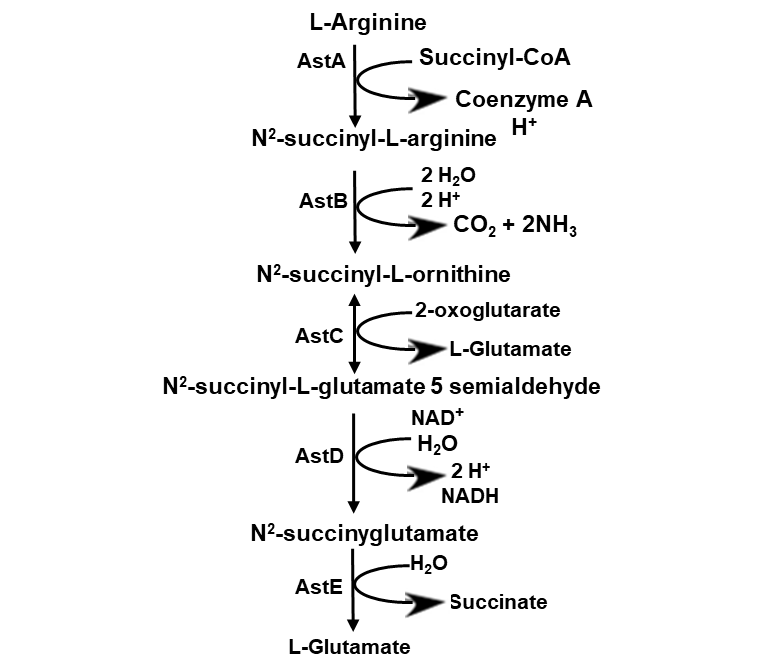


**Figure S4.** Schematic representation of the arginine succinyl transferase pathway in *Acinetobacter baumannii*. The AST pathway in A. baumannii ATCC 17978 VU involves key enzymes that enable the catabolism of arginine as a carbon source (3,7). These include **AstA** (arginine N-succinyl transferase), which initiates the pathway by transferring succinyl-CoA to arginine; **AstB** (N-succinyl arginine dihydrolase), which hydrolyses succinyl arginine into succinyl ornithine; **AstC** (succinyl ornithine transaminase), which catalyses a transamination reaction to produce N-succinyl glutamate; **AstD** (succinyl glutamic semialdehyde dehydrogenase), which reduces succinyl glutamate semialdehyde to succinyl glutamate; and **AstE** (succinyl glutamate desuccinylase), which hydrolyses succinyl glutamate into succinate and glutamate. These enzymes are organized in a single operon (astCADBE), like the organization found in P. aeruginosa and Escherichia coli, highlighting the functional conservation of this pathway.

**
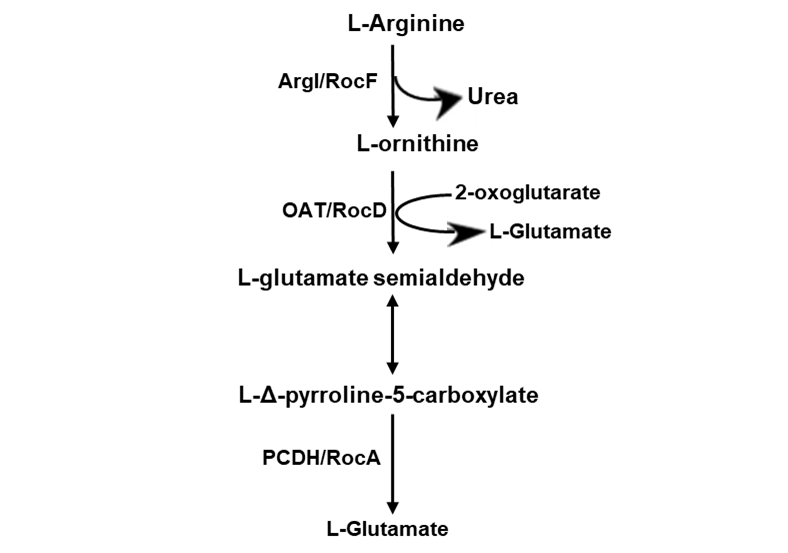
**

**Figure S5.** Schematic representation of the arginase pathway. Arginase (ArgI or RocF) hydrolyses arginine to urea and ornithine in the initial step of the arginase pathway (8,9). In the second step ornithine and 2-oxoglutarate is converted to glutamate and glutamate semialdehyde by Ornithine amino transferase (OAT or RocD). The cyclization of glutamate semialdehyde to l-Δ-pyrroline-5-carboxylate (P5C) occurs spontaneously and almost entirely. P5C dehydrogenase (PCDH or RocA) transforms P5C into a second glutamate molecule


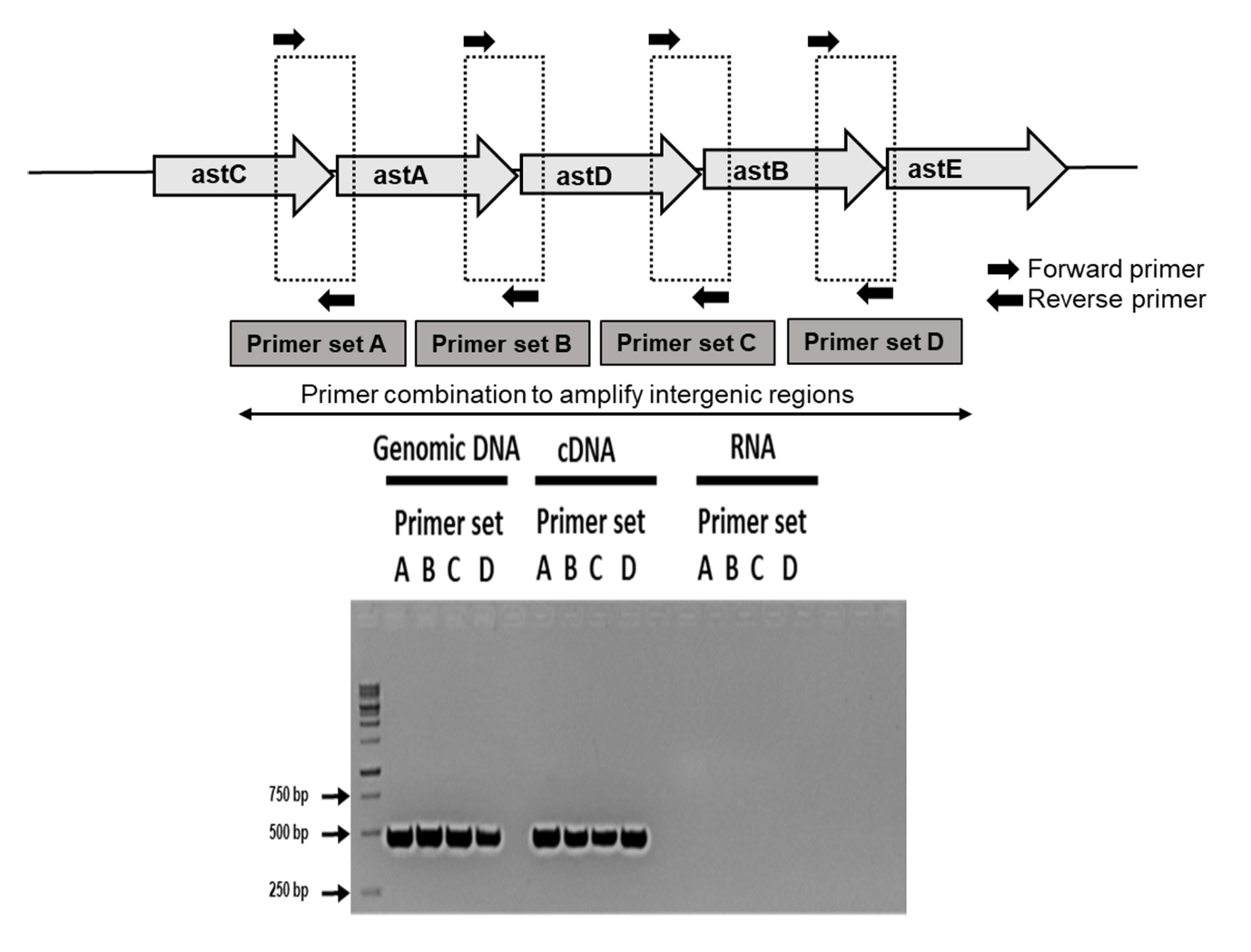


**Figure S6**. The arginine succinyl transferase pathway in *A. baumannii* is an operon. Schematic representation of *astCADBE* locus in *A. baumannii* to investigate the transcriptional linkages between the *astCADBE* genes. Primer binding locations between two genes are marked by the dot lines. Primers sets A, B, C, D are designed to amplify 500 bp of the intergenic region. The forward primer binds to a region 250 bp upstream ahead of the first gene's stop codon, whereas the reverse primer binds to a region 250 bp downstream of second gene's start codon. Images of agarose gel electrophoresis using genomic DNA (positive control), cDNA (Synthesized by reverse transcription of RNA) and RNA (negative control i.e. DNase1 treated RNA) respectively as templates for polymerase chain reactions.  The primer pairs that were used is shown above the gel and a 1 kb DNA marker in first lane was used to determine the amplicon size in the gel. The results show that 500 bp amplicon with the tested primer sets which reveals that the *astCADBE* genes are linked.


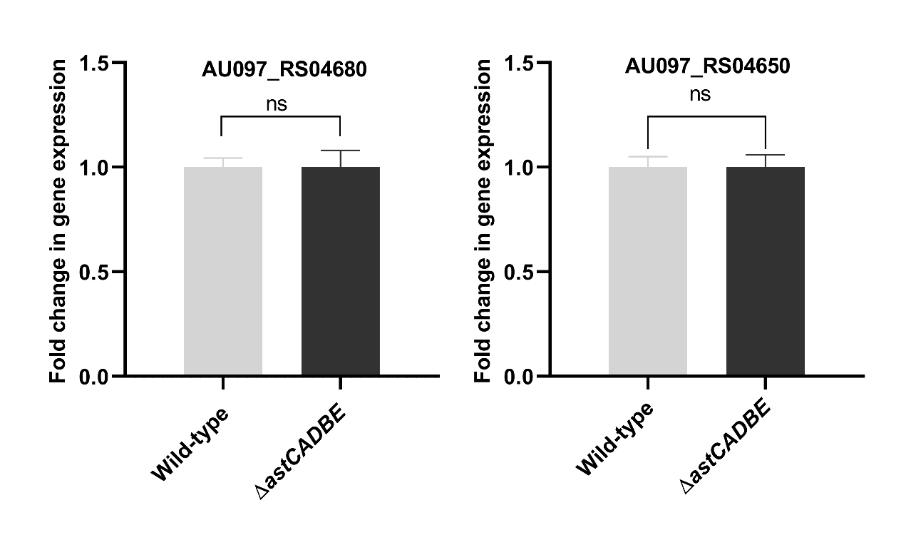


**Figure S7.** Real time PCR (RT-PCR) analysis to determine differences in gene expression of *astC* upstream gene (AU097_RS04680) and *astE* downstream gene (AU097_RS04650) in wild-type and the Δ*astCADBE* strains. To investigate polar effects in the deletion mutant of *astCADBE*, RNA was extracted from both the wild-type and the *astCADBE* mutant, followed by cDNA synthesis. The cDNA was subjected to real-time PCR on a Quant Studio 5 system real-time thermocycler (Applied Biosystems). The target genes AU097_RS04680 and AU097_RS04650 was normalized with the expression of *rpoB* gene and compared. The expression of AU097_RS04680 and AU097_RS04695 showed no difference between wild-type and the Δ*astCADBE* mutant which suggest no polar effects. A student’s *t*-test analysis showed that the fold change in gene expression is non-significant (ns).


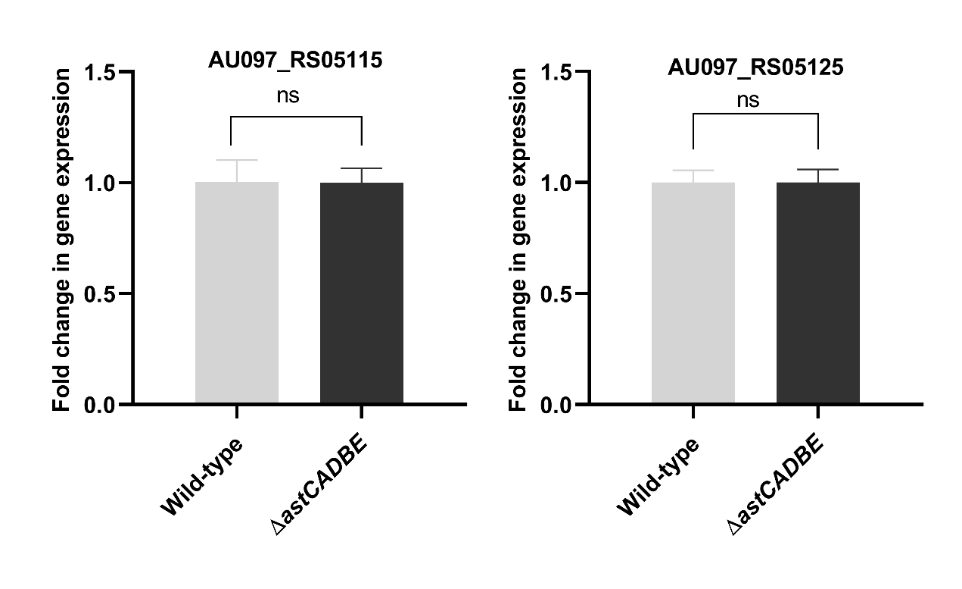


**Figure S8.** RT-PCR analysis to determine differences in gene expression of *argR* upstream gene (AU097_RS05115) and *argR* downstream gene (AU097_RS05125) in wild-type and the Δ*argR* strain. To assess potential polar effects in the *argR* deletion mutant, RNA was extracted from both wild-type and the *argR* mutant. Following RNA extraction, cDNA was synthesized and analysed using real-time PCR on a QuantStudio 5 Real-Time Thermocycler (Applied Biosystems). Gene expression was normalized to the *rpoB* gene and compared between samples. No polar effect was observed in the upstream or downstream of *argR* genes. A student’s *t*-test analysis showed that the fold change in gene expression is non-significant (ns).


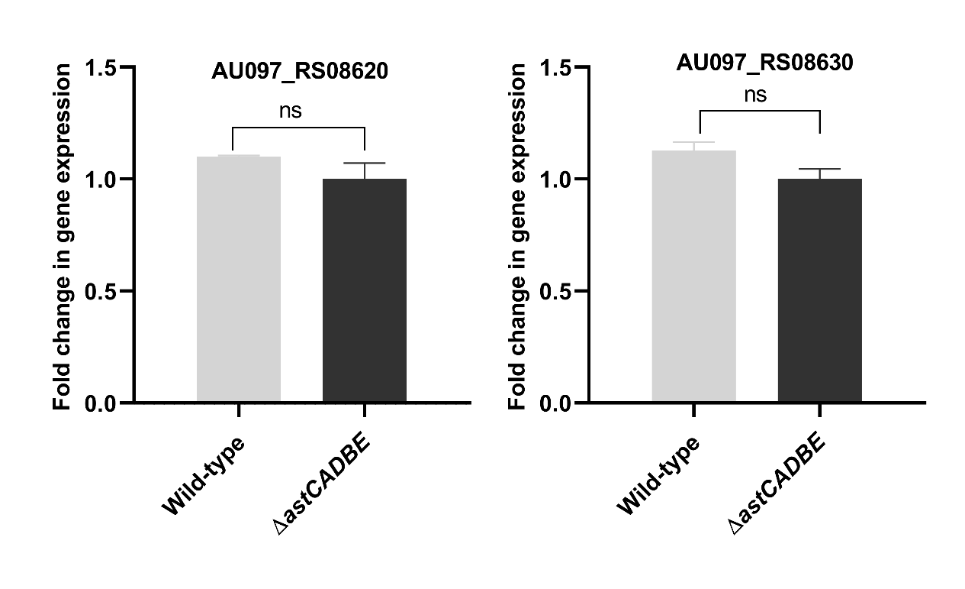


**Figure S9.** RT-PCR analysis to determine differences in gene expression of gacA upstream gene (AU097_RS08620) and downstream gene (AU097_RS08630) in wild-type and ΔgacA strain. To examine potential polar effects of the gacA deletion, RNA was extracted from wild-type and the gacA mutant. The RNA was converted to cDNA and analysed using a QuantStudio 5 Real-Time Thermocycler (Applied Biosystems). Gene expression was normalized to the rpoB gene and compared between the strains. No polar effects were detected in the upstream or downstream gacA genes. A student’s *t*-test analysis confirmed that fold changes in gene expression were not statistically significant (ns).


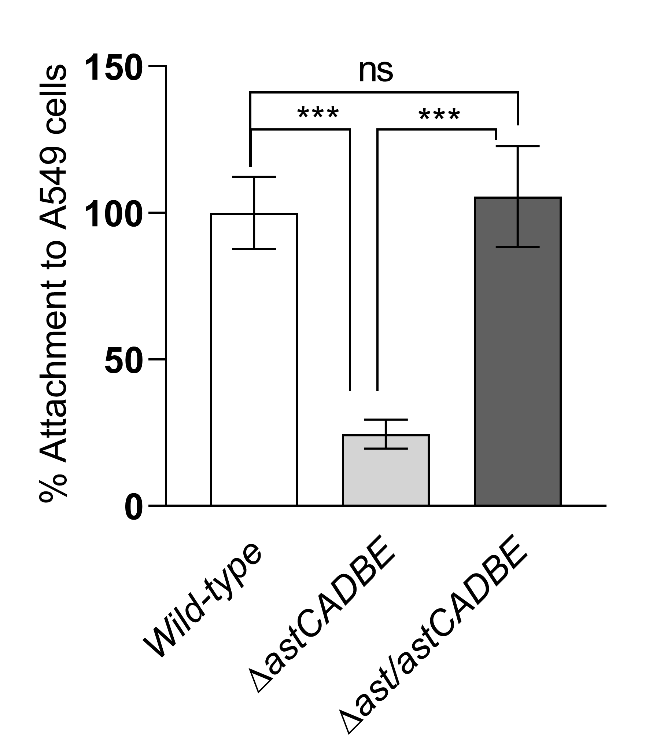


**Figure S10.** The *astCADBE* operon genes are important for attachment to A549 lung epithelial cells. Compared to the wild-type and Δ*ast/astCADBE* strain, the Δ*astCADBE* knockout strain exhibits reduced adhesion to A549 lung epithelial cells. The asterisk (*) denotes statistical significance which was determined using a one-way ANOVA test along with Dunnett's multiple comparison test. The *** denotes a *p*-value of < 0.001, and ns indicates no significance.

**Supplementary Tables**

**Table S1**. List of the bacterial strains that were used in the current study.

| Sl. No. | Strain | Characteristics | Source/references |
| --- | --- | --- | --- |
| 1 | *Acinetobacter baumannii*  ATCC 17978 VU | Wild-type strain | Purchased from  ATCC |
| 2 | *Acinetobacter baumannii*  Δ*astCADBE* | AST pathway knockout | This study |
| 3 | *Acinetobacter baumannii*  Δ*argR* | Response regulator *argR* knockout | This study |
| 4 | *Acinetobacter baumannii*  Δ*gacA* | Response regulator *gacA* knockout | This study |
| 5 | *Acinetobacter baumannii*  Δ*astCADBE/astCADBE* | Genome complementation of *astCADBE* operon in Δ*astCADBE* strain | This study |
| 6 | *Acinetobacter*  *baylyi* | Type strain | Dr. Naveen Kumar Nawani, IIT Roorkee, India |
| 7 | *Acinetobacter baumannii*  AB5075 | Type strain | Purchased from University of Washington (USA) |
| 8 | *Acinetobacter baumannii*  AYE | Type strain | Purchased from  ATCC |
| 9 | *E. coli* BL21 DE3 | *hsdS* gal (cIts857 ind1 Sam7 nin5 lacUV5- T7 gene 1) | Invitrogen, USA |
| 10 | *E. coli* DH5α | supE44 hsdR17 recA1 endA1 gyrA96 thi-1 relA1 | Invitrogen, USA |

**Table S2**. List of the bacterial plasmids that were used in the current study.

| Sl. No. | Plasmid | Characteristics | Source/references |
| --- | --- | --- | --- |
| 1 | pUC18 | Cloning vector in *E. coli*; amp | Thermo Scientific, USA |
| 2 | pMDIAI | Template for apramycin cassette with FRT sites on both sides | Addgene |
| 3 | pAT02 | Plasmid expressing *A. baumannii* RecT homolog, AmpR | Dr. Bryan Davies, University of Texas, Austin, Texas, USA (10). |
| 4 | pAT03 | Plasmid expressing FLP recombinase enzyme (flippase) for expression in *A. baumannii*, AmpR | Dr. Bryan Davies, University of Texas, Austin, Texas, USA (10). |
| 5 | pWBAD30 | Plasmid used for complementation of *argR* and *gacA* gene in *A. baumannii* | Lab stock |

**Table S3**. List of primers that were used in the current study.

| Sl No. | Oligonucleotide | Sequence | Purpose |
| --- | --- | --- | --- |
| 1 | 16s-RNA-RT-FP | ACTTTAAGCGAGGAGGAGGC | qRT-PCR of housekeeping gene  forward primer |
| 2 | 16s-RNA-RT-FP | ATTAACGCTCGCACCCTCTG | qRT-PCR of housekeeping gene forward primer |
| 3 | abUN_ClsC2_FP | GTGATAAAGACAAAAAATTG | Forward primer to determine the presence of Abal44 locus |
| 4 | abUN_ClsC2_RP | ATAAGGTTCCCCACAAAATC | Reverse primer to determine the presence of Abal44 locus |
| 5 | *aruH*-RT-FP | TTACGTCGTCATCCGCAAGT | qRT-PCR of *aruH* gene forward primer |
| 6 | *aruH*-RT-RP | GCATAGCCAATACGCCAACC | qRT-PCR of *aruH* gene reverse primer |
| 7 | *aruI*-RT-FP | TGCCAGCTTTGCTTGGTAGT | qRT-PCR of *aruI* gene forward primer |
| 8 | *aruI*-RT-RP | TAGGCAGCGTTTTCACCCAT | qRT-PCR of *aruI* gene reverse primer |
| 9 | *kauB*-RT-FP | CGCTGCTGGCTCTACACTAA | qRT-PCR of *kauB* gene forward primer |
| 10 | *kauB*-RT-RP | TCGCCATGACTGCAATGTCT | qRT-PCR of *kauB* gene reverse primer |
| 11 | *astC*-RT-FP | ACTGATAAGTTCGCACCGCA | qRT-PCR of *astC* gene forward primer |
| 12 | *astC*-RT-RP | ATGACGGTGTTTCACGCCTT | qRT-PCR of *astC* gene reverse primer |
| 13 | *astA*-RT-FP | TTTGGAATGCGGTAGGCCAT | qRT-PCR of *astA* gene forward primer |
| 14 | *astA*-RT-RP | CTTTCGCATCATCAGGCAGC | qRT-PCR of *astA* gene reverse primer |
| 15 | *astD*-RT-FP | CTGGTTACAAGGCAGCGGTA | qRT-PCR of *astD* gene forward primer |
| 16 | *astD*-RT-RP | TTCTACAATCGCCACACGCT | qRT-PCR of *astD* gene reverse primer |
| 17 | *astB*-RT-FP | TCGCGGTAAGTAACCAGCAG | qRT-PCR of *astB* gene forward primer |
| 18 | *astB*-RT-RP | GTCACACGGTTTTCAGGCAC | qRT-PCR of *astB* gene reverse primer |
| 19 | *astE*-RT-FP | CCGTATGTTCTGTGGTGGGT | qRT-PCR of *astE* gene forward primer |
| 20 | *astE*-RT-RP | GAAGCCCGAATTGCTGTGTG | qRT-PCR of *astE* gene reverse primer |
| 21 | UP500-FP-*astC* | ATTAGTCGACGAAAAACTTAAAGCTAAGTTG | Forward primer for amplification of 500 bp US of *astC* |
| 22 | UP500-RP-*astC* | ATTAGGATCCGCTTACCTCAGTATCTATTTAA | Reverse primer for amplification of 500 bp US of *astC* |
| 23 | DS500-FP-*astE* | ATTAGGTACCATAGTTAATTTTAGGCAAATAAAAAAGGGC | Forward primer for amplification of 500 bp US of *astE* |
| 24 | DS500-RP-*astE* | ATTAGAATTCACCGAAGCAGCTACAGGTAC | Reverse primer for amplification of 500 bp US of *astE* |
| 25 | FP150-Ast-ko | GGCTAATCTGAGGAAACTACCTTTTAG | Forward primer for amplification of *astC* with 150 bp overhang |
| 26 | RP150-Ast-ko | AAATGCAAACGTAAATGCAGGCG | Reverse primer for amplification of *astC* with 150 bp overhang |
| 27 | AprF-Bam | ATCAGGATCCGTCGACCTGCAGTTC | Forward primer for cloning of apramycin gene and FRT into pUC18 |
| 28 | AprR-Kpn | ATGGTACCGTGTAGGCTGGAGCTGCTTC | Reverse primer for cloning of apramycin gene and FRT into pUC18 |
| 29 | RT-FP-AU097_RS04680 | GACCGTGTAGCGCCATACTT | qRT-PCR of *astC* upstream gene forward primer |
| 30 | RT-RP-AU097_RS04680 | CTGGATTGAACCGTCGTCCA | qRT-PCR of *astC* upstream gene reverse primer |
| 31 | RT-FP-AU097_RS04650 | TTAAATGCAGCTCAACCGGC | qRT-PCR of *astE* downstream gene forward primer |
| 32 | RT-RP-AU097_RS04650 | TCGCCTTTTTGTGTAGTCGC | qRT-PCR of *astE* downstream gene reverse primer |
| 33 | *ast*-CA-FP | TGAATATGCAGGTAAAGCG | Forward primer to investigate the transcriptional linkage of *astC* and *astA* gene |
| 34 | *ast*-CA-RP | TTTTGAAGCGTGTACTTGTG | Reverse primer to investigate the transcriptional linkage of *astC* and *astA* gene |
| 35 | *ast*-AD-FP | CAAAATTACCAATGAGGCAACAG | Forward primer to investigate the transcriptional linkage of *astA* and *astD* gene |
| 36 | *ast*-AD-RP | TGTTTCCCAAAAAGGTTTACTGG | Reverse primer to investigate the transcriptional linkage of *astA* and *astD* gene |
| 37 | *ast*-DB-FP | TGATGACTTACTCATTGAAGCACG | Forward primer to investigate the transcriptional linkage of *astD* and *astB* gene |
| 38 | *ast*-DB-RP | CACTAATGTCATCTCCGGTAAAACC | Reverse primer to investigate the transcriptional linkage of *astD* and *astB* gene |
| 39 | *ast*-BE-FP | TGTTTACGTTTACGTGTAGCATTAAATG | Forward primer to investigate the transcriptional linkage of *astB* and *astE* gene |
| 40 | *ast*-BE-RP | ATAATCTCACCGCAAGCTTTAAACG | Reverse primer to investigate the transcriptional linkage of *astB* and *astE* gene |
| 41 | UP500-FP-*argR* | ATTAGTCGAC ATAACCCACAGTATCACGGATTGG | Forward primer for amplification of 500 bp US of *argR* |
| 42 | UP500-RP-*argR* | ATTAGGATCCGAAGACCTCCTTTCTTTC | Reverse primer for amplification of 500 bp US of *argR* |
| 43 | DS500-FP-*argR* | ATTAGGTACCCCAAACTTACATGCAATTTCCC | Forward primer for amplification of 500 bp US of *argR* |
| 44 | DS500-RP-*argR* | ATTAGAATTCAGAAAGCTAAGAGAGCGCTATTACC | Reverse primer for amplification of 500 bp US of *argR* |
| 45 | FP150-*argR*-ko | AGATATAAGGTATAAAGCTTTTACCG | Forward primer for amplification of *argR* with 150 bp overhang |
| 46 | RP150-*argR*-ko | CTGCAAACTGTTATCAGTTTGC | Reverse primer for amplification of *argR* with 150 bp overhang |
| 47 | UP500-FP-*gacA* | ATTAGTCGAC ATAACCCACAGTATCACGGATTGG | Forward primer for amplification of 500 bp US of *gacA* |
| 48 | UP500-RP-*gacA* | ATTAGGATCCGAAGACCTCCTTTCTTTC | Reverse primer for amplification of 500 bp US of *gacA* |
| 49 | DS500-FP- *gacA* | ATTAGGTACCCCAAACTTACATGCAATTTCCC | Forward primer for amplification of 500 bp US of *gacA* |
| 50 | DS500-RP- *gacA* | ATTAGAATTCAGAAAGCTAAGAGAGCGCTATTACC | Reverse primer for amplification of 500 bp US of *gacA* |
| 51 | FP150- *gacA* -ko | AGATATAAGGTATAAAGCTTTTACCG | Forward primer for amplification of *gacA* with 150 bp overhang |
| 52 | RP150-*gacA* -ko | CTGCAAACTGTTATCAGTTTGC | Reverse primer for amplification of *gacA* with 150 bp overhang |
| 53 | RT-FP-AUO97_RS08620 | GTGCAACACCTTTTGGTCCC | qRT-PCR of *gacA* upstream gene forward primer |
| 54 | RT-RP-AUO97_RS08620 | TTGCTGAGAGTTGCCCTAGC | qRT-PCR of *gacA* downstream gene reverse primer |
| 55 | RT-FP-AUO97_RS08630 | AGCGCGTGCTTTAGGTATGA | qRT-PCR of *gacA* downstream gene forward primer |
| 56 | RT-RP-AUO97_RS08630 | GGTCGGTGTGGTTGAGAACT | qRT-PCR of *gacA* downstream gene reverse primer |
| 57 | RT-FP-AUO97_RS05115 | GGCGCCGATATCGTCATACA | qRT-PCR of *argR* upstream gene forward primer |
| 58 | RT-RP-AUO97_RS05115 | ATGATAGCTTGGGTCTGGCG | qRT-PCR of *argR* upstream gene reverse primer |
| 59 | RT-FP-AUO97_RS05125 | GCACTTCAACGGGTGGGATA | qRT-PCR of *argR* downstream gene forward primer |
| 60 | RT-RP-AUO97_RS05125 | TGGTGACCATTTGCTCTCGG | qRT-PCR of *argR* downstream gene reverse primer |
| 61 | FP-*gacA*-comp | ATTAGAATTCCGCAATGTTGTGGTTACAGAAG | Forward primer for amplification of *gacA* gene. |
| 62 | RP-*gacA*-comp | ATTAGTCGACTGCAAACTGTTATCAGTTTGC | Reverse primer for amplification of *gacA* gene. |
| 63 | FP-*argR*-comp | ATTAGAATTCCCATCTCTAGCCCAATTGAAG | Forward primer for amplification of *argR* gene. |
| 64 | RP-argR-comp | ATTAGTCGACACCTCATTGCCGATTTAGAAC | Reverse primer for amplification of *argR* gene. |

**Supplementary methods**

**S1. Identification of genes involved in the arginine decarboxylase pathway in *A. baumannii* ATCC 17978 VU.**

To identify genes associated with the arginine decarboxylase (ADC) pathway in A. baumannii ATCC 17978, a systematic bioinformatics approach was done. The first gene, speA, in the arginine decarboxylase pathway was retrieved from Escherichia coli and Pseudomonas aeruginosa, and an NCBI protein BLAST search was performed. The results showed no similar protein in *A. baumannii* ATCC 1978 like *E. coli* but in contrast showed a protein with a 24.27 % similarity. The gene is annotated as diaminopimelate decarboxylase, the conserved domain search tool of NCBI revealed that the gene is involve in lysine biosynthesis pathway. There are two identified pathways in bacteria that turn agmatine into putrescine. Agmatinase (*speB*) is involved in *E. coli* that catalyses the direct conversion of agmatine to putrescine. Agmatine is converted to putrescine by two enzymes in a second pathway found in *Yersinia pestis* and *P. aeruginosa* where it is converted to N-carbamoyl putrescine by the enzyme aguminase (*aguA*), and then to putrescine by N-carbamoyl putrescine amidohydrolase (*aguB*) (1,2,3,11). The *speB* gene sequence was also retrieved from *E. coli* an NCBI protein BLAST was performed. The results showed no similar protein in *A. baumannii* ATCC 1978 like *E. coli.* The N-carbamoyl putrescine amidohydrolase *aguB* gene was similarly retrieved from *Yersinia pestis* and *P. aeruginosa* further NCBI protein BLAST was performed. The results showed no similar protein in *A. baumannii* ATCC 1978. Hence the arginine decarboxylase (ADC) pathway is not present in *A. baumannii* ATCC 1978.

**S2. Identification of genes involved in the arginine deiminase pathway in *A. baumannii* ATCC 17978 VU.**

To identify genes associated with the arginine deiminase (ADI) pathway in A. baumannii ATCC 17978 VU. The ADI pathway is well studied in *P. aeruginosa* and *Streptococcus pyogenes* (4,5). The arginine deiminase ArcA protein sequence was retrieved from *P. aeruginosa* and *Streptococcus pyogenes* further NCBI protein BLAST was performed. The results showed no similar protein in *A. baumannii* ATCC 1978. The ornithine trans-carbamoylase protein sequence ArcB was also retrieved from *P. aeruginosa* and *S. pyogenes* further NCBI protein BLAST was performed. Both the protein sequences results from the *P. aeruginosa* and *Streptococcus pyogenes* revealed a common protein (NCBI Reference Sequence: WP_001207983.1) annotated as ornithine trans-carbamylase with 34.78 and 33.33 % protein similarity respectively. Though the *arcB* gene is annotated as ornithine trans-carbamylase the conserved domain search tool of NCBI revealed that the gene is involve in arginine biosynthesis pathway and not catabolism.

**S3. Identification of genes involved in the arginine transaminase pathway in *A. baumannii* ATCC 17978 VU.**

The ATA pathway is well characterized in *P. aeruginosa* (1,6). The first enzyme in the ATA pathway, arginine transaminase (AruH), is a key pyridoxal phosphate (PLP)-dependent enzyme essential for arginine catabolism. The *P. aeruginosa* AruH sequence was retrieved and analysed using NCBI protein BLAST, revealing a similar protein in *A. baumannii* ATCC (34.96 % similarity) annotated as a PLP-dependent aminotransferase (locus tag AUO97_RS01430). Conserved domain analysis confirmed its PLP dependency.

The second enzyme in the ATA pathway, AruI, catalyses the decarboxylation of 2-ketoarginine, releasing 4-guanidinobutyraldehyde and CO₂. The P. aeruginosa AruI sequence was retrieved and analysed using NCBI protein BLAST, showing similar proteins in A. baumannii ATCC with 32.28 % and 24.64 % similarity, annotated as acetolactate synthase 3 large subunit (locus tag AUO97_RS10140) and thiamine pyrophosphate-binding protein (locus tag AUO97_RS01135), respectively. Conserved domain analysis indicates the first protein is the catalytic subunit of acetolactate synthase, involved in branched-chain amino acid synthesis, while the second protein belongs to the alpha-keto acid decarboxylase family, suggesting potential decarboxylase activity. These findings indicate the presence of an AruI-like protein in A. baumannii ATCC 17978. Unlike in P. aeruginosa PAO1, Pseudomonas putida, and Pseudomonas fluorescens, where the genes for AruH and AruI are in a single operon (1), A. baumannii ATCC 17978 VU has these genes in separate locations.

In the third step, the enzyme 4-guanidinobutyraldehyde dehydrogenase (KauB) oxidizes 4-guanidinobutyraldehyde to 4-guanidinobutyrate, transferring electrons to NAD^+^ to form NADH. The P. aeruginosa KauB sequence was retrieved and analysed via NCBI protein BLAST, revealing a similar protein in A. baumannii ATCC with 40.54 % similarity, annotated as an NAD^+^ dependent aldehyde dehydrogenase (locus tag AUO97_RS18630).

In the fourth step, the GbuA enzyme encodes guanidino butyrase, which converts 4-guanidinobutyrate to 4-aminobutyrate. GbuA protein sequence from P. aeruginosa PAO1 was analysed via NCBI BLAST, revealing a similar protein in A. baumannii ATCC 17978 VU (25.32 % similarity), annotated as the *hutG* gene (locus tag AUO97_RS07720). HutG, involved in the histidine utilization pathway, functions as N-formyl glutamate amidohydrolase, converting N-formyl-L-glutamate to L-glutamate and formate. Conserved domain analysis indicates the protein is a metalloenzyme requiring Mn²⁺ for its catalytic activity. The results showed no similar protein in A. baumannii ATCC 17978 VU. In P. aeruginosa PAO1, an AST pathway mutant can grow on arginine if functional ATA genes are present. However, when the AST mutant also has a disrupted gbuA gene, growth on arginine is abolished (1).

In the fifth step of the ATA pathway, 4-aminobutyrate transaminase (GabT) catalyses the reaction between 4-aminobutyrate and 2-oxoglutarate, producing glutamate and succinate semialdehyde. The P. aeruginosa PAO1 GabT sequence was retrieved and analysed via NCBI BLAST, revealing a similar protein in A. baumannii ATCC 17978 VU with 55.16 % similarity, annotated as 4-aminobutyrate-2-oxoglutarate transaminase (locus tag AUO97_RS05455). Conserved domain analysis suggests its function in transamination.

The sixth enzyme of ATA pathway, GabD utilizes NAD+ as a cofactor to oxidize succinate semialdehyde to succinate, linking arginine catabolism to the tricarboxylic acid cycle. Protein sequence analysis of GabD from *P. aeruginosa* revealed a homologous protein in *A. baumannii* ATCC 17978 VU, showing 33.12 % similarity and annotated as an aldehyde dehydrogenase family protein (locus tag AUO97_RS01145). The conserved domain analysis suggests similarity to *Fusarium fujikuroi* aldehyde dehydrogenase FUS7, essential for fusarin C biosynthesis. The results showed no similar protein in *A. baumannii* ATCC 17978 VU.

**S4. Identification of genes involved in the arginine succinyl transferase pathway in *A. baumannii* ATCC 17978 VU.**

Protein sequences for AST pathway enzymes were retrieved from NCBI using P. aeruginosa templates. NCBI BLAST and conserved domain searches confirm the presence of these enzymes in A. baumannii ATCC 17978 VU. The pathway enzymes were found to share significant functional similarity with those in P. aeruginosa. These enzymes include AstA (N-succinyl transferase) with 48.96 % similarity, AstB (N-succinyl arginine dihydrolase) with 61.43 % similarity, AstC (succinyl ornithine transaminase) with 61.15 % similarity, AstD (succinyl glutamic semialdehyde dehydrogenase) with 61.43 % similarity, and AstE (succinyl glutamate desuccinylase) with 38.41 % similarity. The genetic organization of these enzymes in A. baumannii ATCC 17978 VU is consistent with P. aeruginosa and *E. coli* with all five genes arranged in a single operon, astCADBE (3,7).

**S5. Identification of genes involved in the arginase pathway in *A. baumannii* ATCC 17978 VU.**

The arginase pathway is well studied in Bacillus and Helicobacter *species* (8,9). The arginase protein sequence was retrieved from Bacillus and Helicobacter *species.* NCBI BLAST and domain searches revealing a similar protein in A. baumannii ATCC 17978 VU, annotated as *hutG* (locus tag AUO97_RS07720), involved in histidine catabolism, not arginine. In the second step of the arginase pathway, ornithine aminotransferase (OAT) catalyses the conversion of ornithine and 2-oxoglutarate to glutamate. Using similar approach like above we found proteins of similar function from the aspartate aminotransferase family, such as glutamate-1-semialdehyde 2,1-aminomutase and succinyl ornithine transaminase, involved in transamination or decarboxylation. For the third step protein sequences from Bacillus and Helicobacter *spp.* were retrieved, further NCBI BLAST and conserved domain analysis identified a bifunctional proline dehydrogenase/L-glutamate gamma-semialdehyde dehydrogenase in *A. baumannii* ATCC 17978 VU, which oxidizes proline to glutamate and acts as a transcriptional repressor. These findings suggest that A. baumannii ATCC 17978 VU lacks the key arginase enzyme, and thus does not utilize the arginase pathway.

**Supplementary references.**

1. Yang, Zhe, and Chung-Dar Lu. "Functional genomics enables identification of genes of the arginine transaminase pathway in *Pseudomonas aeruginosa*." *Journal of bacteriology* 189, no. 11 (2007): 3945-3953.

2. Charlier, D. and Bervoets, I., 2019. Regulation of arginine biosynthesis, catabolism and transport in *Escherichia coli*. *Amino acids*, 51, pp.1103-1127.

3. Schneider, Barbara L., Alexandros K. Kiupakis, and Lawrence J. Reitzer. "Arginine catabolism and the arginine succinyl transferase pathway in *Escherichia coli*." *Journal of bacteriology* 180, no. 16 (1998): 4278-4286.

4. Nakada, Yuji, and Yoshifumi Itoh. "Characterization and regulation of the *gbuA* gene, encoding guanidino butyrase in the arginine dehydrogenase pathway of *Pseudomonas aeruginosa* PAO1." *Journal of bacteriology* 184, no. 12 (2002): 3377-3384.

5. Cusumano, Zachary T., Michael E. Watson Jr, and Michael G. Caparon. "*Streptococcus pyogenes* arginine and citrulline catabolism promotes infection and modulates innate immunity." *Infection and immunity* 82, no. 1 (2014): 233-242.

6. Yang, Zhe, and Chung-Dar Lu. "Characterization of an arginine: pyruvate transaminase in arginine catabolism of *Pseudomonas aeruginosa* PAO1." *Journal of bacteriology* 189, no. 11 (2007): 3954-3959.

7. Itoh, Yoshifumi. (1997). Cloning and characterization of the *aru* genes encoding enzymes of the catabolic arginine succinyl transferase pathway in *Pseudomonas aeruginosa*. *Journal of bacteriology*, *179*(23), 7280-7290.

8. Xiong, Lifeng, Jade LL Teng, Michael G. Botelho, Regina C. Lo, Susanna KP Lau, and Patrick CY Woo. "Arginine metabolism in bacterial pathogenesis and cancer therapy." *International journal of molecular sciences* 17, no. 3 (2016): 363.

9. Hernández, Victor M., Alejandra Arteaga, and Michael F. Dunn. "Diversity, properties and functions of bacterial arginases." *FEMS Microbiology Reviews* 45, no. 6 (2021): fuab034.

10. Tucker, A.T., Nowicki, E.M., Boll, J.M., Knauf, G.A., Burdis, N.C., Trent, M.S. and Davies, B.W., 2014. Defining gene-phenotype relationships in *Acinetobacter baumannii* through one-step chromosomal gene inactivation. *MBio*, *5*(4), pp.10-1128.

11. Hampel, Annegret, Claudia Huber, Robert Geffers, Marina Spona-Friedl, Wolfgang Eisenreich, and Franz-Christoph Bange. "*Mycobacterium tuberculosis* is a natural ornithine aminotransferase (rocD) mutant and depends on Rv2323c for growth on arginine." *PLoS One* 10, no. 9 (2015): e0136914.
